# The novel SMYD3 inhibitor EM127 impairs DNA repair response to chemotherapy-induced DNA damage and reverses cancer chemoresistance

**DOI:** 10.1186/s13046-024-03078-9

**Published:** 2024-05-30

**Authors:** Paola Sanese, Katia De Marco, Martina Lepore Signorile, Francesca La Rocca, Giovanna Forte, Marialaura Latrofa, Candida Fasano, Vittoria Disciglio, Elisabetta Di Nicola, Antonino Pantaleo, Giusy Bianco, Vito Spilotro, Claudia Ferroni, Matilde Tubertini, Nicoletta Labarile, Lucia De Marinis, Raffaele Armentano, Gianluigi Gigante, Valerio Lantone, Giuliano Lantone, Marina Naldi, Manuela Bartolini, Greta Varchi, Alberto Del Rio, Valentina Grossi, Cristiano Simone

**Affiliations:** 1 Medical Genetics, National Institute of Gastroenterology, IRCCS “Saverio de Bellis” Research Hospital, Castellana Grotte (Ba), 70013 Italy; 2 Animal Facility, National Institute of Gastroenterology, IRCCS “Saverio de Bellis” Research Hospital, Castellana Grotte (Ba), 70013 Italy; 3 Institute of Organic Synthesis and Photoreactivity - National Research Council, Bologna, 40129 Italy; 4 Department of Chemical and Environmental Sciences, University of Insubria, Como, 22100 Italy; 5 Histopathology Unit, National Institute of Gastroenterology, IRCCS “Saverio de Bellis” Research Hospital, Castellana Grotte (Ba), 70013 Italy; 6 General Surgery Unit, National Institute of Gastroenterology, IRCCS “Saverio de Bellis” Research Hospital, Castellana Grotte (Ba), 70013 Italy; 7 General Surgery Unit, Department of Precision and Regenerative Medicine and Jonic Area (DiMePRe-J), University of Bari Aldo Moro, Bari, 70124 Italy; 8 Unit of Surgery, “Lorenzo Bonomo” Hospital, Andria, BAT Italy; 9 Department of Pharmacy and Biotechnology, Alma Mater Studiorum University of Bologna, Bologna, 40126 Italy; 10 Innovamol Consulting Srl, Modena, 41126 Italy; 11 Medical Genetics, Department of Precision and Regenerative Medicine and Jonic Area (DiMePRe-J), University of Bari Aldo Moro, Bari, 70124 Italy

**Keywords:** Cancer, SMYD3, Drug resistance, Chemosensitivity, DNA damage response

## Abstract

**Background:**

SMYD3 has been found implicated in cancer progression. Its overexpression correlates with cancer growth and invasion, especially in gastrointestinal tumors. SMYD3 transactivates multiple oncogenic mechanisms, favoring cancer development. Moreover, it was recently shown that SMYD3 is required for DNA restoration by promoting homologous recombination (HR) repair.

**Methods:**

In cellulo and in vivo models were employed to investigate the role of SMYD3 in cancer chemoresistance. Analyses of SMYD3-KO cells, drug-resistant cancer cell lines, patients’ residual gastric or rectal tumors that were resected after neoadjuvant therapy and mice models were performed. In addition, the novel SMYD3 covalent inhibitor EM127 was used to evaluate the impact of manipulating SMYD3 activity on the sensitization of cancer cell lines, tumorspheres and cancer murine models to chemotherapeutics (CHTs).

**Results:**

Here we report that SMYD3 mediates cancer cell sensitivity to CHTs. Indeed, cancer cells lacking SMYD3 functions showed increased responsiveness to CHTs, while restoring its expression promoted chemoresistance. Specifically, SMYD3 is essential for the repair of CHT-induced double-strand breaks as it methylates the upstream sensor ATM and allows HR cascade propagation through CHK2 and p53 phosphorylation, thereby promoting cancer cell survival. SMYD3 inhibition with the novel compound EM127 showed a synergistic effect with CHTs in colorectal, gastric, and breast cancer cells, tumorspheres, and preclinical colorectal cancer models.

**Conclusions:**

Overall, our results show that targeting SMYD3 may be an effective therapeutic strategy to overcome chemoresistance.

**Supplementary Information:**

The online version contains supplementary material available at 10.1186/s13046-024-03078-9.

## Introduction

Cancer treatment is constantly evolving. Currently, the main therapeutic strategy is chemotherapy, an aggressive form of chemical drug therapy that destroys rapidly growing cells by damaging DNA. The typical DNA damage induced by chemotherapy comprises double-strand breaks (DSBs), which are the most toxic DNA lesions but also the most effective in triggering DNA damage response (DDR) [1]. During the S/G2 phase of the cell cycle, DSBs are mainly corrected by the homologous recombination (HR) pathway, which ensures accurate repair [2]. In particular, the upstream sensor ATM promotes HR-mediated DNA repair by activating RAD51 to process DSBs [3]. Unfortunately, the efficacy of chemotherapy is limited by the toxicity due to non-specific effects on normal tissues and by the development of chemoresistance, i.e., the occurrence of molecular changes that make cancer cells insensitive to a particular drug [4].

In this context, new approaches are being developed. Better outcomes and decreased side effects have been achieved by combining chemotherapy with targeted therapy, which consists of the use of drugs that can block cancer growth and spread by interfering with specific molecules involved in these processes [5]. Therefore, an attractive therapeutic strategy is to target factors involved in DSB repair activation, which is one of the main mechanisms that promote chemoresistance [6]; indeed, interesting results have been obtained by targeting ATM, ATR, and RAD51 [7–10]. Both genetic and epigenetic alterations may be responsible for poor treatment response in cancer cells by inducing the activation of molecular mechanisms involved in chemoresistance [11–14], thereby contributing to the selection of cells with a resistant phenotype [15]. Thus, targeting epigenetic modifiers is a promising therapeutic approach as it can lead to the re-sensitization of tumors to chemotherapy due to the reversibility of epigenetic abnormalities.

The methyltransferase SMYD3 is a regulator of epigenetic and signaling pathways in cancer and has drawn significant attention from researchers and companies focusing on the identification and targeting of novel factors to develop effective treatment strategies. This is due to the correlation observed between SMYD3 overexpression and cancer cell growth in several types of tumors and to SMYD3 oncogenic activity as a transcriptional activator of genes and co-regulator of pathways involved in transformation and cancer progression [16, 17]. Based on this evidence, SMYD3 is emerging as a potential risk and prognostic factor.

In a previous study, we performed a comprehensive in silico analysis to cluster all potential SMYD3-interacting proteins identified by screening the human proteome for a library of rare tripeptides, based on their involvement in cancer hallmarks. This approach led to the identification of new SMYD3 interactors involved in processes related to cancer hallmarks [18, 19]. Recent studies on SMYD3 oncogenic role also revealed that it can be crucial for unperturbed cell division by promoting phase transition and allowing cancer cells to bypass cell cycle arrest signals [16]. Moreover, we recently showed that SMYD3 has a protective role during cell response to genotoxic stress by promoting the restoration of damaged DNA via the HR repair pathway, thereby sustaining cancer cell genomic stability and tumor progression [20, 21]. Consistently, combined inhibition of SMYD3 and PARP, which is one of the most studied DDR targets, emerged as a promising synthetic lethality strategy in HR-proficient gastrointestinal and breast cancers expressing high levels of SMYD3 [20].

Identifying the main players involved in the complex network of signaling pathways that govern cancer development and chemoresistance, together with a full comprehension of their mechanisms of action, is crucial for designing new approaches to re-sensitize cancer cells to common therapies. In this light, based on novel evidence on the role of SMYD3 as a cancer genome keeper, we investigated in depth its involvement in drug resistance mechanisms. Our results show that SMYD3 activity mediates DDR in response to chemotherapeutics (CHTs) and that its inhibition with the covalent inhibitor EM127 [22] sensitizes cancer cells to chemotherapy. These findings support the potential of this combination treatment to overcome resistance.

## Results

### SMYD3 is a promising molecular target to sensitize cancer cells to chemotherapy

**Fig. 1:**
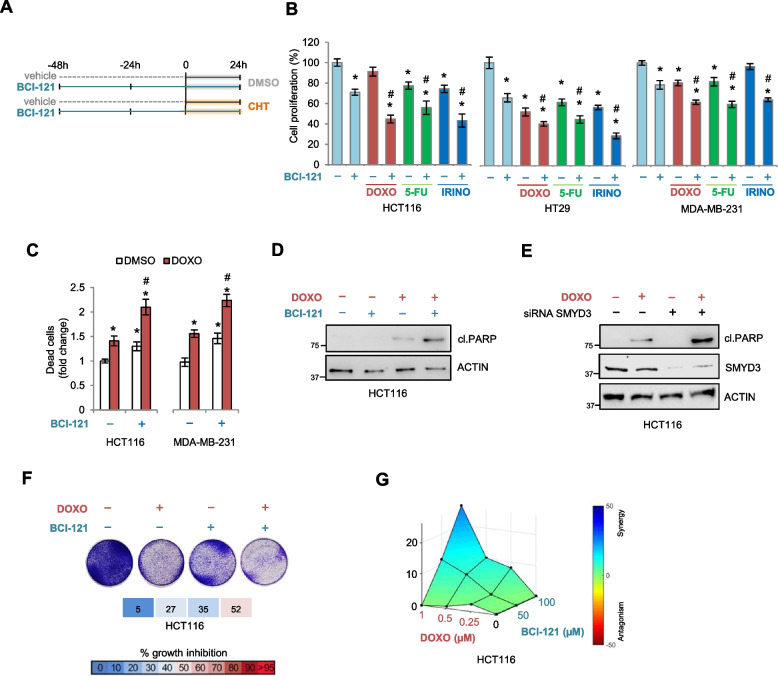
SMYD3 inhibition with BCI-121 sensitizes cancer cells to chemotherapeutics. **A** Treatment scheme: HCT116, HT29, and MDA-MB-231 cells were pre-treated or not with BCI-121 (100 μM) for 48 h and then treated or not with 5-fluorouracil (10 μM), doxorubicin (1 μM), or irinotecan (10 μM) for another 24 h in the presence of BCI-121. **B** Quantification of cell proliferation by CellTiter 96 Aqueous Assay in HCT116, HT29, and MDA-MB-231 cells treated as described in (**A**). **C** Quantification of cell death by trypan blue staining in HCT116 and MDA-MB-231 cells treated with BCI-121 and/or doxorubicin as described in (**A**). **D** Immunoblot analysis of cleaved PARP in HCT116 cells treated with BCI-121 and/or doxorubicin as described in (**A**). ACTIN was used as a loading control. **E** Immunoblot analysis of cleaved PARP in HCT116 transfected with siRNAs against SMYD3 for 48 h and then treated or not with doxorubicin (1 μM) for 24 h. ACTIN was used as a loading control. **F** Colony formation assay of HCT116 cells treated with BCI-121 and/or doxorubicin as described in (**A**). **G** Bliss synergy surface analysis obtained with Combenefit software of HCT116 cells treated with different concentrations of BCI-121 (0, 50, 100 μM) and doxorubicin (0, 0.25, 0.5, 1 μM). *p < 0.05 treated vs. untreated. #*p* < 0.05 combined treatment vs. single treatments. cl.PARP = cleaved PARP; DMSO = dimethyl sulfoxide; DOXO = doxorubicin; 5-FU = 5-fluorouracil; IRINO = irinotecan

Recently, SMYD3 has been shown to play an important role in the regulation of DNA damage checkpoint dynamics in cancer cells by inducing the formation of HR complexes and promoting HR repair in response to genotoxic stress, thereby allowing DSB restoration and hence cancer cell genomic stability [16, 20]. We thus investigated whether SMYD3 inhibition could improve the effects of conventional DSB-inducing CHTs to devise new options for rational and effective combination therapies. To this aim, we first employed the largely-used SMYD3 inhibitor (SMYD3i) BCI-121, which we previously identified and characterized [23]. We focused on potential changes in the sensitivity of HCT116 and HT29 colorectal cancer (CRC) and MDA-MB-231 breast cancer (BC) cells pre-treated with BCI-121 to short-term exposure to DSB-inducing CHTs (Fig. 1A). Our results showed that BCI-121 increases the effect of CHTs on cancer cell proliferation (Fig. 1B). We further analyzed the biological impact of this approach by characterizing changes in cell fate. Our data revealed that combined treatment with doxorubicin and BCI-121 increases apoptosis (Fig. 1C, D). Moreover, we confirmed that the pro-apoptotic effect was dependent upon SMYD3 inhibition, as shown by RNAi-mediated SMYD3 depletion (Fig. 1E). Notably, colony formation assays confirmed the ability of the combined treatment to induce cytotoxicity (Fig. 1F). Finally, the synergistic activity of doxorubicin and BCI-121 was confirmed by the Bliss model (Combenefit) for synergy analysis (Fig. 1G).

### SMYD3 overexpression correlates with chemoresistance in gastrointestinal cancers

**Fig. 2:**
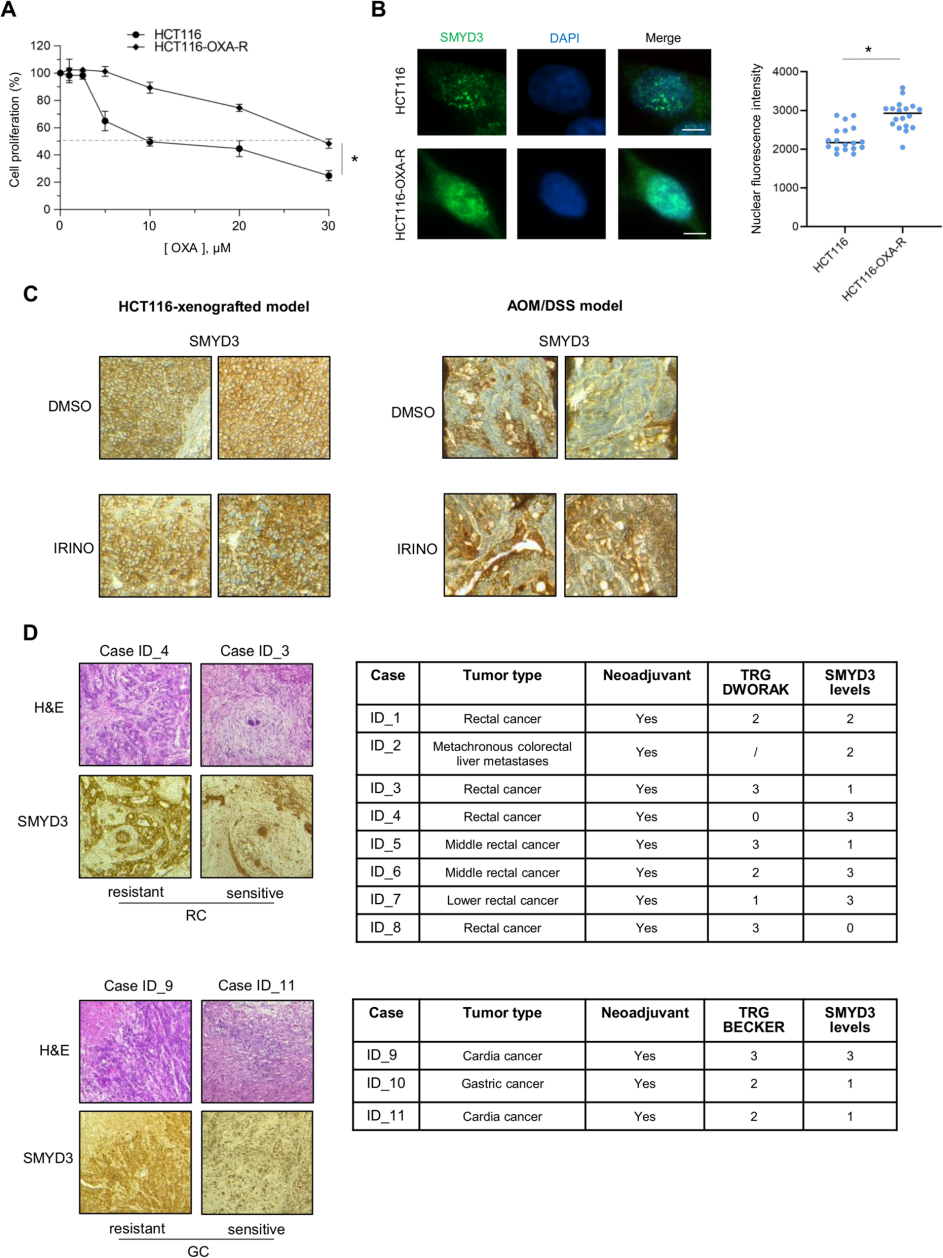
SMYD3 is overexpressed in chemoresistant gastrointestinal cancers. **A** Quantification of cell proliferation by CellTiter 96 Aqueous Assay in HCT116 parental cells and HCT116-OXA-R oxaliplatin-resistant cells in response to 72 h of treatment with different doses of oxaliplatin (0–30 μM). **B** Immunofluorescence analysis of SMYD3 expression in HCT116 and HCT116-OXA-R cells. Nuclei were counterstained with DAPI (blue) (left panel). The graph reflects the SMYD3 nuclear fluorescence intensity detected in the two cell lines (right panel). Scale bar: 5 μm **C** Immunohistochemistry analysis of SMYD3 expression in HCT116-xenografted mice (left panel) and AOM/DSS-treated mice (right panel) injected with irinotecan or the vehicle (DMSO). Two representative samples are displayed. Magnification 20x. **D** Hematoxylin and eosin staining and immunohistochemistry analysis of SMYD3 expression in rectal (RC, upper left panel) and gastric (GC, lower left panel) tumor tissues which are representative of neoadjuvant-resistant and sensitive cases, according to the relative tumor regression grade (Dworak or Becker). Table summary of the analyzed RC (upper right panel) and GC (lower right panel) samples, indicating tumor type, neoadjuvant use, tumor regression grade (Dworak/Becker), and SMYD3 immunoreactivity levels: 0 = absent, 1 = mild and focal, 2 = moderate, 3 = intense and diffuse. *p < 0.05 resistant vs. parental cell line. AOM = azoxymethane; DMSO = dimethyl sulfoxide; DSS = dextran sodium sulfate; GC = gastric cancer; H&E = hematoxylin and eosin; IRINO = irinotecan; OXA = oxaliplatin; RC = rectal cancer; TRG = tumor regression grade

The above evidence prompted us to analyze the role of SMYD3 in cancer cells showing resistance to CHTs. To this end, we generated an oxaliplatin-resistant CRC cell line (HCT116-OXA-R) in order to mimic the acquired chemoresistance mechanisms that can occur in cancer patients during chemotherapy. These cells were three-fold more resistant to oxaliplatin than their parental counterpart (Fig. 2A). Moreover, immunofluorescence staining showed that SMYD3 expression was higher in the nucleus of HCT116-OXA-R cells, suggesting that increased SMYD3 nuclear levels could be involved in chemoresistance (Fig. 2B).

To support this hypothesis, we analyzed SMYD3 expression in two preclinical CRC models, i.e., HCT116-xenografted and AOM/DSS mice, treated or not with irinotecan. Our data revealed an enrichment of SMYD3 in residual tumors that had not undergone complete regression after irinotecan exposure, further suggesting that increased expression of SMYD3 may contribute to the cellular mechanisms that mediate cancer chemoresistance (Fig. 2C).

Based on these data, we carried out an immunohistochemical staining analysis of SMYD3 in 11 patients with gastric (GC) or rectal (RC) cancer treated with neoadjuvant chemotherapy and then subjected to surgical resection. Samples were clustered in two groups based on their responsiveness to the treatment according to Dworak (for RC) or Becker (for GC) tumor regression grading system (Fig. 2D). Our data showed that SMYD3 expression was significantly higher in chemoresistant than in nonresistant rectal and gastric tumors (Fig. 2D). Notably, areas of partial tumor regression showed high SMYD3 expression in the viable part of the residual tumor (Fig. 2D).

### SMYD3 activity is required to promote cancer cell chemoresistance

**Fig. 3:**
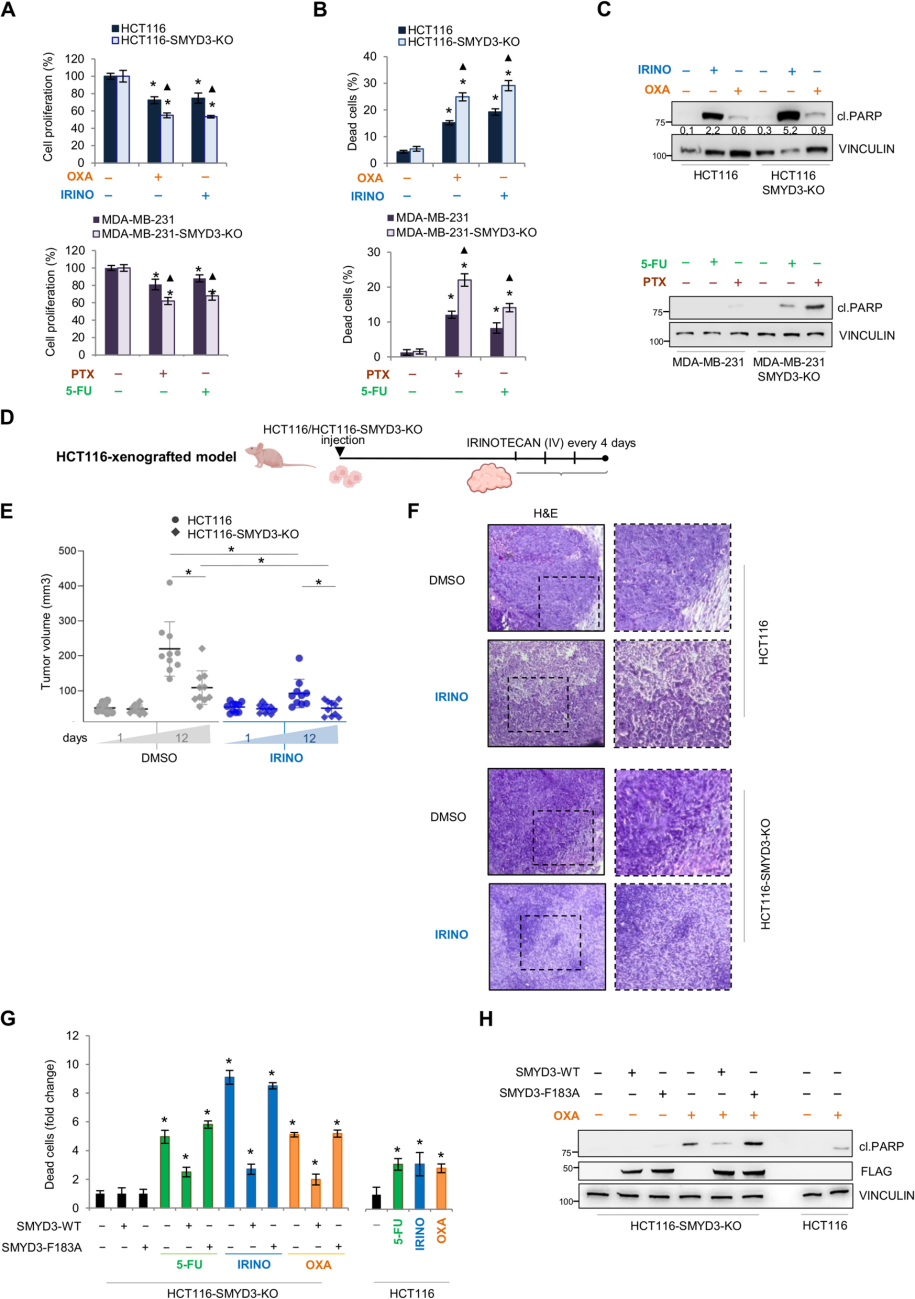
SMYD3 mediates cancer cell chemosensitivity. **A**, **B** Quantification of cell proliferation by CellTiter 96 Aqueous Assay (**A**) and cell death by trypan blue staining (**B**) in HCT116 vs HCT116-SMYD3-KO cells treated with oxaliplatin (10 μM) or irinotecan (10 μM) for 48 h (upper panel) and MDA-MB-231 vs MDA-MB-231-SMYD3-KO cells treated with 5-fluorouracil (10 μM) or paclitaxel (100 nM) for 48 h (lower panels). **C** Immunoblot analysis of cleaved PARP in HCT116 vs HCT116-SMYD3-KO cells treated with oxaliplatin (10 μM) or irinotecan (10 μM) for 48 h (upper panel) and MDA-MB-231 vs MDA-MB-231-SMYD3-KO cells (lower panel) treated with 5-fluorouracil (10 μM) or paclitaxel (100 nM). The numbers on the top panel indicate the densitometric analysis of cleaved PARP intensity normalized to the loading control VINCULIN. **D** Treatment scheme of HCT116-xenografted mice. As soon as the tumors reached a measurable size, mice were treated with irinotecan (20 mg/kg) every 4 days. **E**, **F** Volume quantification (**E**) and hematoxylin and eosin staining (**F**) of tumors explanted from HCT116- or HCT116-SMYD3-KO-xenografted mice treated with the vehicle (DMSO) or irinotecan. Magnification 10-20x. **G** Quantification of cell death by trypan blue staining in HCT116-SMYD3-KO cells transfected with FLAG-SMYD3-WT or FLAG-SMYD3-F183A and treated with 5-fluorouracil (10 μM), irinotecan (10 μM), or oxaliplatin (10 μM) for 24 h, compared with the parental untransfected HCT116 cell line. **H** Immunoblot analysis of cleaved PARP in HCT116-SMYD3-KO cells transfected with FLAG-SMYD3-WT or FLAG-SMYD3-F183A and treated with oxaliplatin (10 μM) for 24 h, compared with the parental untransfected HCT116 cell line. FLAG was analyzed as an overexpression control and VINCULIN was used as a loading control. *p < 0.05 treated vs. untreated; ▲*p* < 0.05 SMYD3-KO treated cells vs. treated parental cells. cl.PARP = cleaved PARP; DMSO = dimethyl sulfoxide; DSS = dextran sodium sulfate; 5-FU = 5-fluorouracil; H&E = hematoxylin and eosin; IRINO = irinotecan; IV = intravenous; OXA = oxaliplatin; PTX = paclitaxel

Next, to validate the direct involvement of SMYD3 in chemotherapy response, we first generated a SMYD3-Knockout HCT116 cell line (HCT116-SMYD3-KO) using the CRISPR/Cas9 system for genome editing. HCT116-SMYD3-KO cells and the previously generated MDA-MB-231-SMYD3-KO [20] cells were exposed to CHTs for 48 h to test their chemosensitivity. Analysis of cell proliferation, cell death, and PARP cleavage showed that CRC and BC cells lacking SMYD3 were more sensitive to CHTs than their parental counterparts (Fig. 3A-C). Then, to evaluate in vivo the effect of knocking out SMYD3 on cancer cell chemosensitivity, we analyzed tumor growth in mice. Xenograft tumors were established by injecting HCT116 or HCT116-SMYD3-KO cells into athymic nude mice. As soon as the tumors reached a measurable size, mice were divided into two groups, which were treated with the vehicle or irinotecan. Drug treatments were administered every 4 days by intravenous injection for 12 days (Fig. 3D), and tumor size and body weight were recorded every 2–3 days. After 12 days, the tumor volume in HCT116-SMYD3-KO-xenografted mice was significantly lower compared to HCT116-xenografted mice (Fig. 3E), in line with previous studies showing that SMYD3 deficiency inhibits tumor development [24, 25]. In particular, irinotecan exposure completely arrested the growth of SMYD3-KO tumors (Fig. 3E). At the end of the treatment, the xenograft tumors were explanted and subjected to histological examination (Fig. 3F). This analysis revealed significant tumor regression, which was mainly characterized by the presence of fibrotic tissue tending to restrain neoplasm invasion in SMYD3-KO tumors treated with irinotecan compared with those treated with the vehicle alone. Interestingly, peripheral necrosis with signs of inflammation was observed in SMYD3-KO tumors, while expansive growth was detected in SMYD3-wild-type (WT) tumors (Fig. 3F). Collectively, these data suggest that SMYD3 plays a crucial role in cancer resistance to CHT treatments in vivo.

Thus, to confirm the direct contribution of SMYD3 in mediating cancer cell responsiveness to genotoxic drugs, we transiently restored SMYD3 expression in HCT116-SMYD3-KO cells with a construct leading to the synthesis of WT SMYD3 (FLAG-SMYD3-WT) or an enzymatic-inactive mutant (FLAG-SMYD3-F183A). We found that restoring the expression of WT SMYD3 reduced cancer cell sensitivity to 5-fluorouracil, irinotecan, and oxaliplatin, resulting in drug tolerance levels comparable to those observed in the parental cell line (Fig. 3G). Conversely, the overexpression of a catalytically inactive form of SMYD3 did not restore chemoresistance, indicating that SMYD3 catalytic activity is essential to promote the development of drug resistance (Fig. 3G). Consistent results were obtained by analyzing cleaved PARP levels to detect apoptosis induction after oxaliplatin exposure (Fig. 3H).

### Blocking SMYD3 with the novel inhibitor EM127 is an effective strategy to overcome chemoresistance in gastrointestinal and breast cancer cells

**Fig. 4:**
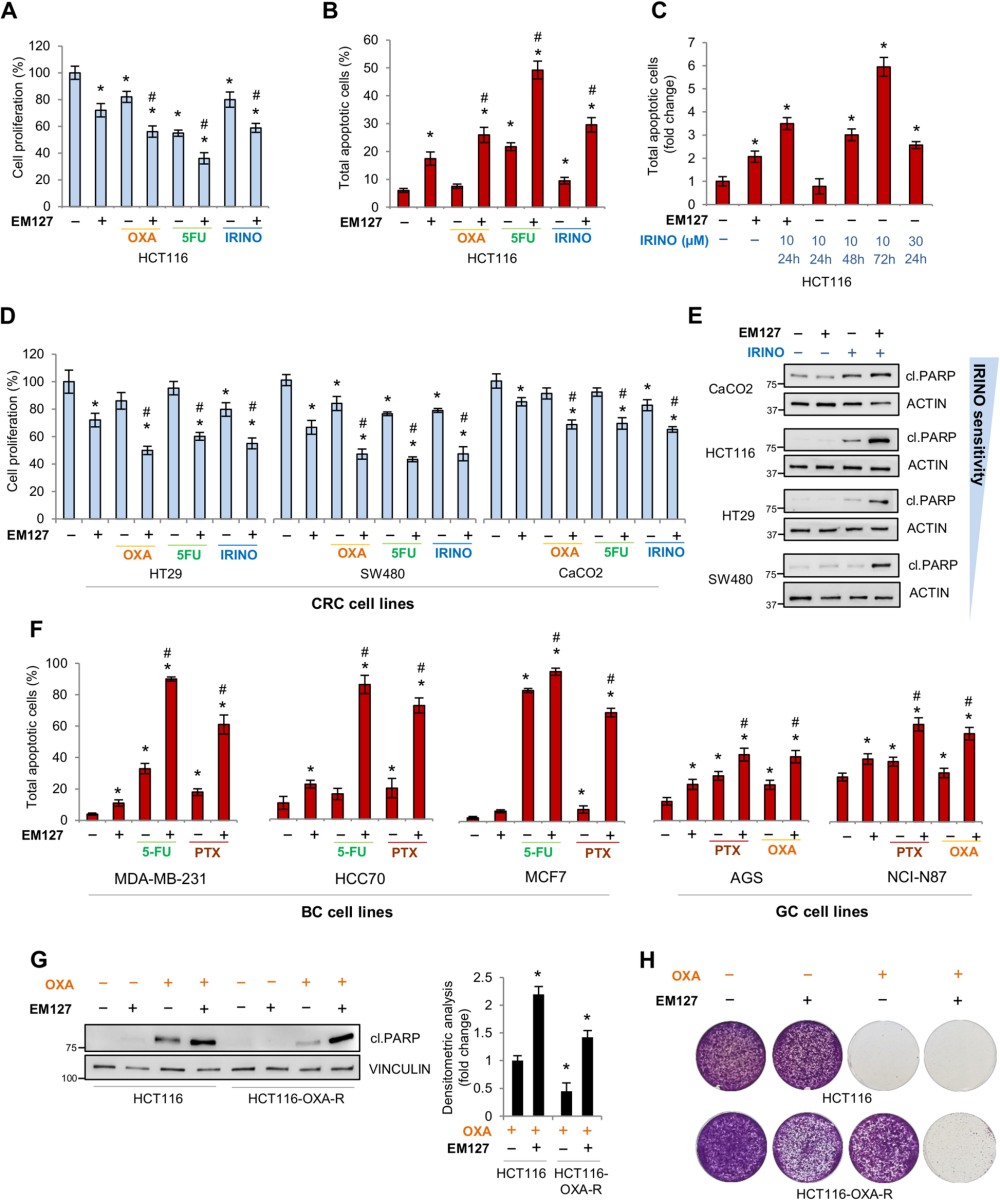
SMYD3 inhibition with EM127 overcomes chemoresistance in gastrointestinal and breast cancer cell lines. **A**, **B** Quantification of cell proliferation by CellTiter 96 Aqueous Assay (**A**) and apoptotic cell death by flow cytometry analysis of Annexin V staining (**B**) in HCT116 cells pre-treated or not with EM127 (5 μM) for 48 h and then treated or not with oxaliplatin (10 μM), 5-fluorouracil (10 μM), or irinotecan (10 μM) for another 24 h in the presence of EM127. **C** Quantification of apoptotic cell death by flow cytometry analysis of Annexin V staining in HCT116 cells treated with different doses (10, 30 μM) of irinotecan for different times (24, 48, 72 h) compared with cells pre-treated with EM127 (5 μM) for 48 h and then treated with irinotecan (10 μM) for 24 h in the presence of EM127. **D** Quantification of cell proliferation by CellTiter 96 Aqueous Assay in HT29, SW480, and CaCO2 cells treated as described in (**A**). **E** Immunoblot analysis of cleaved PARP in CaCO2, HCT116, HT29, and SW480 cells pre-treated or not with EM127 (5 μM) for 48 h and then treated or not with irinotecan (10 μM) for another 24 h in the presence of EM127. ACTIN was used as a loading control. **F** Quantification of apoptotic cell death by flow cytometry analysis of Annexin V staining in MDA-MB-231, HCC70, MCF7, AGS, and NCI-N87 cells pre-treated or not with EM127 (5 μM) for 48 h and then treated or not with 5-fluorouracil (10 μM) or paclitaxel (100 nM) (BC cell lines) or with paclitaxel (10 nM) or oxaliplatin (10 μM) (GC cell lines) for another 24 h in the presence of EM127. **G** Immunoblot analysis of cleaved PARP in HCT116 and HCT116-OXA-R cells pre-treated or not with EM127 (5 μM) for 48 h and then treated or not with oxaliplatin (10 μM) for another 24 h in the presence of EM127. VINCULIN was used as a loading control. The graph on the right showed the densitometric analysis of cleaved PARP intensity normalized to the loading control. **H** Colony formation assay of HCT116 and HCT116-OXA-R cells treated or not with EM127 (5 μM) and/or oxaliplatin (10 μM) for 10 days after plating. *p < 0.05 treated vs. untreated. #*p* < 0.05 combined treatment vs. single treatments. BC = breast cancer; CRC = colorectal cancer; GC = gastric cancer; cl.PARP = cleaved PARP; 5-FU = 5-fluorouracil; IRINO = irinotecan, OXA = oxaliplatin; PTX = paclitaxel

We recently developed a new covalent SMYD3i, named EM127, which can form a stable complex with SMYD3, providing potent inhibition of methyltransferase activity and longer-lasting effects [22]. Thus, we evaluated the efficacy of this novel compound in sensitizing cancer cells to CHTs. We first compared EM127 to the previously characterized SMYD3i BCI-121. We found that EM127 is significantly more effective than BCI-121, as it showed a synergistic effect with doxorubicin at a 20-fold lower dosage (Supplementary Fig. 1A). HCT116 CRC cells were then treated with a combination of EM127 and CHTs that are commonly used for CRC treatment (oxaliplatin, 5-fluorouracil, irinotecan) [26]. Cell proliferation assays revealed that EM127 sensitizes HCT116 cells to CHTs (Fig. 4A). We further evaluated the biological impact of these combined treatments by investigating the induction of apoptosis. This analysis revealed an increase in total apoptotic cells with the combined treatments compared to the administration of each drug alone (Fig. 4B, Supplementary Fig. 1B). Moreover, we found that the time and dose needed for irinotecan to trigger apoptosis decreased when it was administered in combination with EM127 (Fig. 4C, Supplementary Fig. 1B), and their synergistic activity was confirmed by the Bliss model (Supplementary Fig. 1C). The combined treatments were then tested in other CRC cell lines (HT29, SW480, CaCO2) with different genetic backgrounds and drug sensitivity levels, showing that EM127 increases responsiveness to CHTs also in these cells (Fig. 4D, E, Supplementary Fig. 1D).

Since SMYD3 activity has been found critical in other gastrointestinal cancers, such as GC [27–29], and its genetic or pharmacological ablation has already shown to be a promising strategy to arrest BC progression [23, 30, 31], we extended the analysis of EM127 effect on chemosensitivity to GC and BC cell lines. These cells were treated with CHTs that are used specifically for these tumors [32, 33]. Triple-negative breast cancer (TNBC) cell lines (MDA-MB-231, HCC70), which are usually resistant to standard therapy, were also included. Our data showed that EM127 makes all GC and BC cell lines tested more sensitive to CHTs, reversing treatment resistance in chemoresistant cells (Fig. 4F, Supplementary Fig. 1E, F).

In addition, we compared the effect of the combined treatment in HCT116 and HCT116-OXA-R cells. Our results revealed that SMYD3 inhibition increased sensitivity to oxaliplatin in the resistant cell line, as shown by cleaved PARP levels (Fig. 4G). Intriguingly, colony formation assays confirmed that EM127 reversed the acquired chemoresistance in oxaliplatin-resistant cells. Indeed, SMYD3 inhibition had a chemosensitizing effect on HCT116-OXA-R cells, making them unable to form colonies after exposure to oxaliplatin, similar to their parental counterpart (Fig. 4H).

Overall, our data confirmed that SMYD3 inhibition with EM127 is a promising approach to overcome chemoresistance in CRC, GC, and BC.

### SMYD3 regulates CHT-induced ATM-CHK2-p53 cascade activation, thereby mediating HR repair

**Fig. 5:**
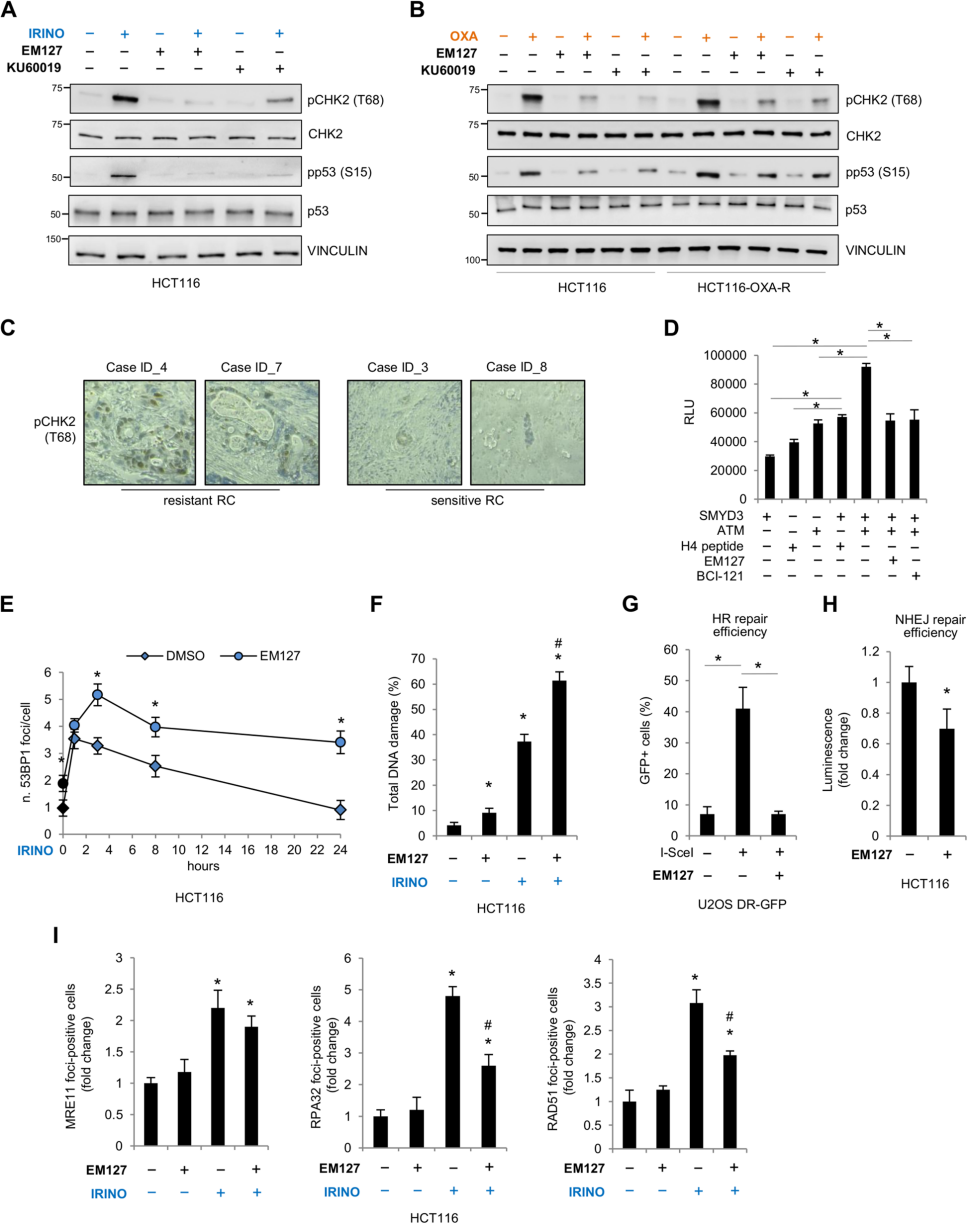
SMYD3 mediates the repair of CHT-induced DNA damage by activating the ATM-CHK2-p53 cascade. **A**, **B** Immunoblot analysis of the phosphorylation levels of CHK2 (at T68) and p53 (at S15) in HCT116 (**A**, **B**) and HCT116-OXA-R (**B**) cells pre-treated or not with EM127 (5 μM) for 25 h or with KU60019 (5 μM) for 24 h and then exposed or not to irinotecan (10 μM) (**A**) or to oxaliplatin (10 μM) (**B**) for 6 h in the presence of the inhibitor. VINCULIN was used as a loading control. **C** Immunohistochemistry analysis of pCHK2 expression in rectal tumor tissues that are representative of neoadjuvant-resistant or neoadjuvant-sensitive cases according to Dworak tumor regression grade (see Fig. 2D). **D** In vitro methylation assay showing ATM methylation by SMYD3, as measured by the luminescence signal resulting from SAH generation. The H4 peptide was used as a control substrate. Methylation activity was also assessed in the presence of SMYD3 inhibitors EM127 (5 μM) or BCI-121 (100 μM). **E** Quantification of 53BP1 foci/cell in HCT116 cells pre-treated or not with EM127 (5 μM) for 2.5 h and then exposed or not to irinotecan (10 μM) and fixed at the indicated time points. 53BP1 foci were quantified by immunofluorescence analysis. *p < 0.05 EM127-treated vs DMSO **F** Analysis of total DNA damage in HCT116 cells pre-treated or not with EM127 (5 μM) for 12 h and then exposed or not to irinotecan (10 μM) for 6 h. Cells were stained for pATM (S1981) and γH2AX (S139) to calculate the percentage of cells with DNA damage. **G** Evaluation of HR repair efficiency. U2OS DR-GFP cells were pre-treated or not with EM127 for 2.5 h and then transfected with a vector encoding for the I-SceI enzyme. After 24 h, HR activity was measured by determining the percentage of GFP+ cells for each condition. **H** Evaluation of NHEJ repair efficiency. HCT116 cells were pre-treated or not with EM127 for 24 h and then transfected with the linearized or uncut pGL3 vector. After 24 h, NHEJ activity was measured by normalizing the luciferase activity of the linearized pGL3 signal to the uncut pGL3 control signal. **I** Quantification of MRE11, RPA32, and RAD51 foci-positive cells in HCT116 cells pre-treated or not with EM127 (5 μM) for 2.5 h and then exposed or not to irinotecan (10 μM) for 6 h. Foci-positive cells were quantified by immunofluorescence analysis. *p < 0.05 treated vs. untreated. #*p* < 0.05 combined treatment vs. single treatments. HR = Homologous recombination; IRINO = irinotecan; NHEJ = Non homologous end joining; OXA = oxaliplatin; RC = rectal cancer; RLU = relative luminescence unit

One of the most important molecular mechanisms of chemoresistance in cancer is the activation of the ATM-CHK2-p53 cascade, which plays a central role in the regulation of HR upon DSBs [3]. We thus analyzed the activation of this signaling cascade in HCT116 cells treated with CHTs used in clinical settings for CRC therapy, such as irinotecan and oxaliplatin, which directly or indirectly induce DSBs [34]. Moreover, since the data presented above indicate that SMYD3 activity is required for drug response and resistance, we tested the effect of EM127 on the HR cascade in these conditions. We found that SMYD3 inhibition reduced ATM-mediated phosphorylation of CHK2 and p53 at levels comparable to those observed with the ATM inhibitor KU60019, demonstrating that SMYD3 is involved in the activation of the ATM-CHK2-p53 cascade (Fig. 5A, B). Intriguingly, similar results were obtained in oxaliplatin-resistant cells, in which the HR pathway is extensively activated to restore oxaliplatin-mediated damage, thereby promoting the resistant phenotype (Fig. 5B). These findings, which were also confirmed in other cell lines (Supplementary Fig. 2A), suggest that SMYD3 enzymatic activity is crucial for DNA repair-mediated chemoresistance and its inhibition impairs the activation of the ATM-CHK2-p53 cascade, allowing to overcome resistance to CHTs. We also investigated whether the combination of SMYD3 and ATM inhibition could enhance the effects observed with each inhibitor alone. We found that combining these drugs at half doses reduced CHK2 and p53 phosphorylation at levels comparable to those observed with the single drugs at full doses, while their combination at full doses enhanced the observed effect (Supplementary Fig. 2B). This combination also showed an additive effect in sensitizing cancer cells to irinotecan (Supplementary Fig. 2C), suggesting that it may be of interest for clinical investigations as a synthetic lethality approach.

Since CHK2 upregulation has been previously detected in tumor tissues that are resistant to genotoxic drug treatment [35], we analyzed CHK2 phosphorylation in human RC samples (see Fig. 2D) as a marker of effective DNA repair and induction of chemoresistance. Immunohistochemical assays showed that CHK2 phosphorylation is upregulated in neoadjuvant chemotherapy-resistant compared to sensitive RC samples (Fig. 5C). These data suggest a positive correlation between CHK2 phosphorylation and SMYD3 expression, adding additional insight into the molecular mechanisms that regulate chemoresistance.

SMYD3 oncogenic activity has been linked to cancer progression through multiple mechanisms, including the interaction with and methylation of non-histone proteins [16]. We recently showed that SMYD3 can bind to ATM, favoring the formation of HR complexes during DSB response [20]. These findings suggest that SMYD3 could exert its role as a methyltransferase on ATM, the HR upstream sensor kinase, thereby promoting the activation of this pathway. To verify this hypothesis, we performed an in vitro methylation assay using purified proteins. Our results showed that SMYD3 can efficiently methylate ATM, and this modification is efficiently inhibited by both EM127 and BCI-121 (Fig. 5D). These data indicate that SMYD3 may regulate the HR pathway by exerting its methyltransferase activity on the upstream ATM kinase.

To assess whether SMYD3 inhibition affects HR-mediated repair of DSBs caused by irinotecan exposure, we then evaluated the DNA repair process by detecting nuclear damage foci. Analysis of the DSB marker 53BP1 revealed the presence of a higher number of 53BP1 foci in HCT116 cells treated with EM127 (Fig. 5E, Supplementary Fig. 2D, 0 h time point), suggesting that this novel SMYD3 covalent inhibitor increases the amount of unrepaired endogenous DSB lesions, as previously reported with the reversible inhibitor BCI-121 in BC cells [20]. Next, we evaluated the ability of EM127-pre-treated HCT116 cells to repair irinotecan-induced DNA damage. We found that after treatment with EM127, HCT116 cells failed to reduce the increased amount of 53BP1 foci for up to 24 h of drug exposure (Fig. 5E, Supplementary Fig. 2D). Moreover, the total DNA damage induced by irinotecan increased in EM127-pre-treated HCT116 cells, indicating that SMYD3 inhibition prevents efficient repair and resolution of DSBs (Fig. 5F, Supplementary Fig. 2E). In order to get further insight into this mechanism and assess the effect of EM127 on the efficiency of the HR and non-homologous end joining (NHEJ) repair pathways, we performed HR- and plasmid-based end-joining assays using U2OS DR-GFP cells, transfected with a vector encoding for the I-SceI restriction enzyme, and HCT116 cells transfected with a linearized pGL3 vector, respectively. Our results revealed that SMYD3 inhibition hampered the HR repair process (Fig. 5G). These data confirm previous findings [20, 36] and are consistent with our observations in cancer cells with CHT-induced DSBs, which are primarily recognized and repaired by the HR pathway [34]. Analysis of NHEJ efficiency revealed that EM127 treatment also seems to affect the NHEJ repair process (Fig. 5H), consistent with recent findings in reporter cells treated with BCI-121 [37]. Analysis of MRE11 foci formation showed that SMYD3 inhibition did not significantly affect MRE11 recruitment to damaged sites (Fig. 5I, Supplementary Fig. 2F). Upon DNA damage, MRE11, along with RAD50 and NBS1, forms the MRN complex, which is essential for activating the HR pathway upstream of ATM [38]. This finding suggests that SMYD3 is involved in DSB repair by directly modulating ATM activity rather than affecting other upstream factors. Immunofluorescence analysis of RPA32 punctate nuclear foci, which are an indicator of actively ongoing HR repair [39], and of foci containing RAD51, which is a critical HR effector and is loaded onto RPA32 coated single-stranded DNA at DSBs [40], further confirmed that EM127 impaired the recruitment of those proteins to irinotecan-induced DSBs (Fig. 5I, Supplementary Fig. 2F).

Overall, these data confirm that SMYD3 inhibition leads to the failure of proper repair of CHT-induced DNA damage.

### Targeting SMYD3 in preclinical models to circumvent chemoresistance

**Fig. 6:**
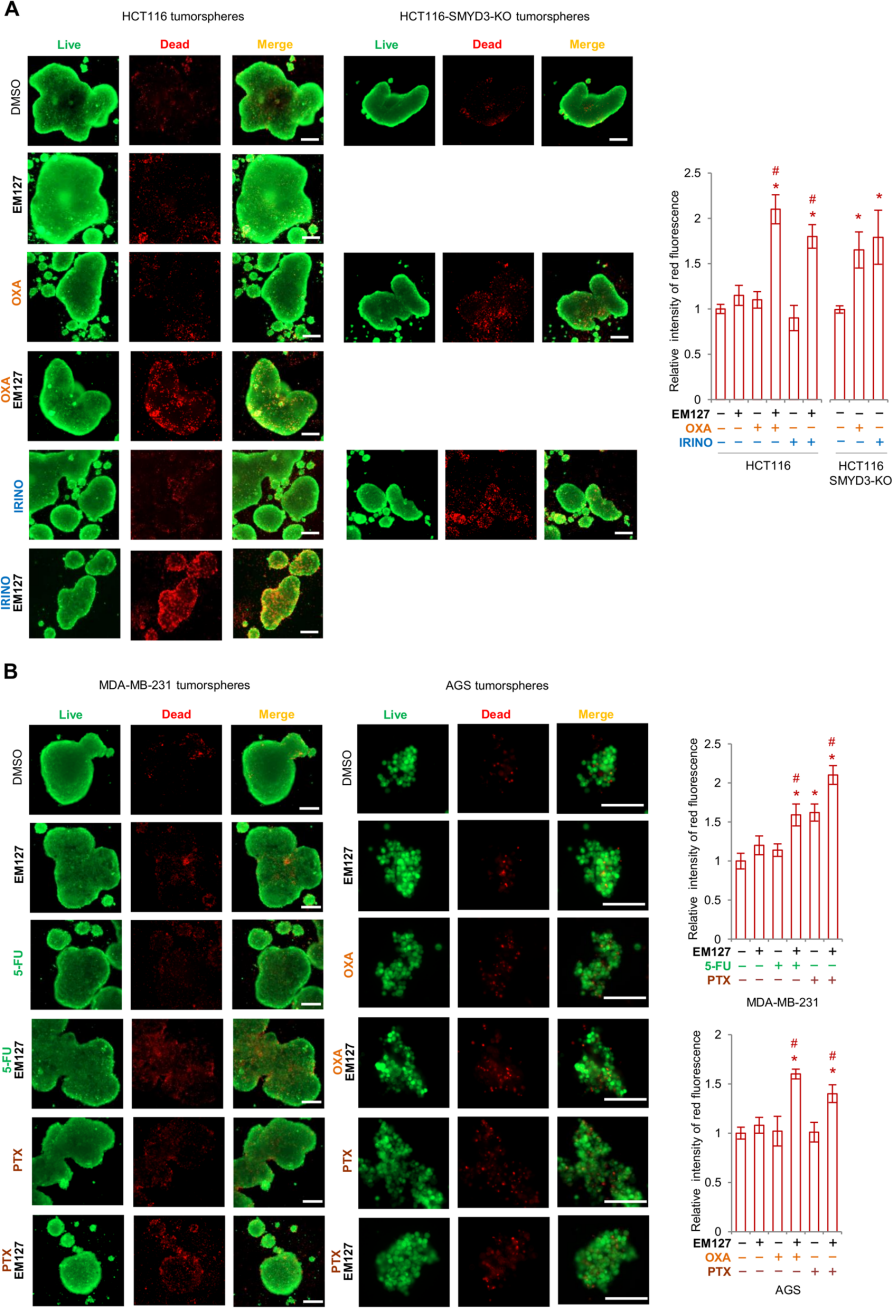
SMYD3 inhibition promotes CRC, BC, and GC tumorspheres chemosensitization. **A** Live/dead staining of HCT116 and HCT116-SMYD3-KO cells grown as 3D tumorspheres. HCT116 cells were pre-treated or not with EM127 for 48 h and then treated or not with irinotecan (10 μM) or oxaliplatin (10 μM) for another 24 h in the presence of EM127. HCT116-SMYD3-KO cells were treated or not with irinotecan (10 μM) or oxaliplatin (10 μM) for 24 h. The graph on the right shows the relative intensity of dead cell staining (red fluorescence). **B** Live/dead staining of MDA-MB-231 and AGS cells grown as 3D tumorspheres, pre-treated or not with EM127 for 48 h and then treated or not with 5-fluorouracil (5 μM) or paclitaxel (100 nM) (MDA-MB-231) or with oxaliplatin (20 μM) or paclitaxel (10 nM) (AGS) for another 24 h in the presence of EM127. The graph on the right shows the mean intensity of dead cell staining (red fluorescence). Scale bar: 200 μm. *p < 0.05 treated vs. untreated. #*p* < 0.05 combined treatment vs. single treatments. DMSO = dimethyl sulfoxide; 5-FU = 5-fluorouracil; IRINO = irinotecan; OXA = oxaliplatin; PTX = paclitaxel

In order to lay the groundwork for future potential clinical applications of SMYD3i/CHT combined therapies, we tested the effect of EM127 in preclinical cancer models, such as three-dimensional (3D) cell cultures and AOM/DSS mice. 3D cell cultures are emerging as a powerful research tool that mimics in vivo cell environments; moreover, it has been shown that non-adherent 3D spheroids display greater resistance to CHTs compared to bidimensional cell cultures [41]. Based on these premises, we generated tumorspheres from the HCT116 CRC cell line and its SMYD3-KO counterpart to evaluate the effect of SMYD3 ablation or pharmacological inhibition on cancer cell proliferation and chemotherapy response in these more complex systems. A live/dead staining assay performed on HCT116 and HCT116-SMYD3-KO tumorspheres showed that knocking out SMYD3 reverses the chemoresistant phenotype of HCT116 tumorspheres (Fig. 6A). Consistently, pre-treatment with EM127 for 48 h sensitized HCT116 tumorspheres to oxaliplatin and irinotecan, causing a marked reduction in cell survival and increased cell death (Fig. 6A). These data confirmed the synergistic effect of the SMYD3i/CHT combined treatment.

We then evaluated this approach in mammospheres and gastrospheres generated from MDA-MB-231 BC and AGS GC cells, respectively, by combining EM127 with CHTs commonly used for BC and GC treatment (Fig. 6B). Overall, the data collected on tumor spheroids strongly support the efficacy of SMYD3 inhibition in increasing the cytotoxic effects of CHTs and therefore its potential as a therapeutic strategy to overcome chemoresistance.

Next, we performed in vivo experiments in the AOM/DSS colitis-associated murine carcinoma model, which is considered a highly reproducible system that recapitulates human CRC [42]. C57BL/6 mice were administered with the carcinogen azoxymethane (AOM), followed by three cycles of dextran sulfate sodium (DSS) to induce colitis. To increase EM127 solubility and bioavailability, we took advantage of nanoformulation technologies, which have exhibited great potential in significantly improving the stability and the pharmacokinetic and pharmacodynamic profiles of various bioactives [43]. In this context, human serum albumin (HSA), the major serum protein, represents a validated and safe biomaterial that can extend therapeutics half-life and preferentially accumulates within the tumor mass, thereby reducing off-target side-effects [44]. To this end, we encapsulated EM127 within HSA-based nanoparticles (EM127@HSA) by exploiting an in-water drug-induced aggregation procedure [45]. In vitro stability studies were performed by monitoring particle size changes under different conditions by dynamic light scattering (DLS, see supporting information) and revealed no significant changes in hydrodynamic diameter and polydispersity values over a week, confirming the outstanding colloidal stability of these nanoparticles (Supplementary Fig. 3A, B).

**Fig. 7:**
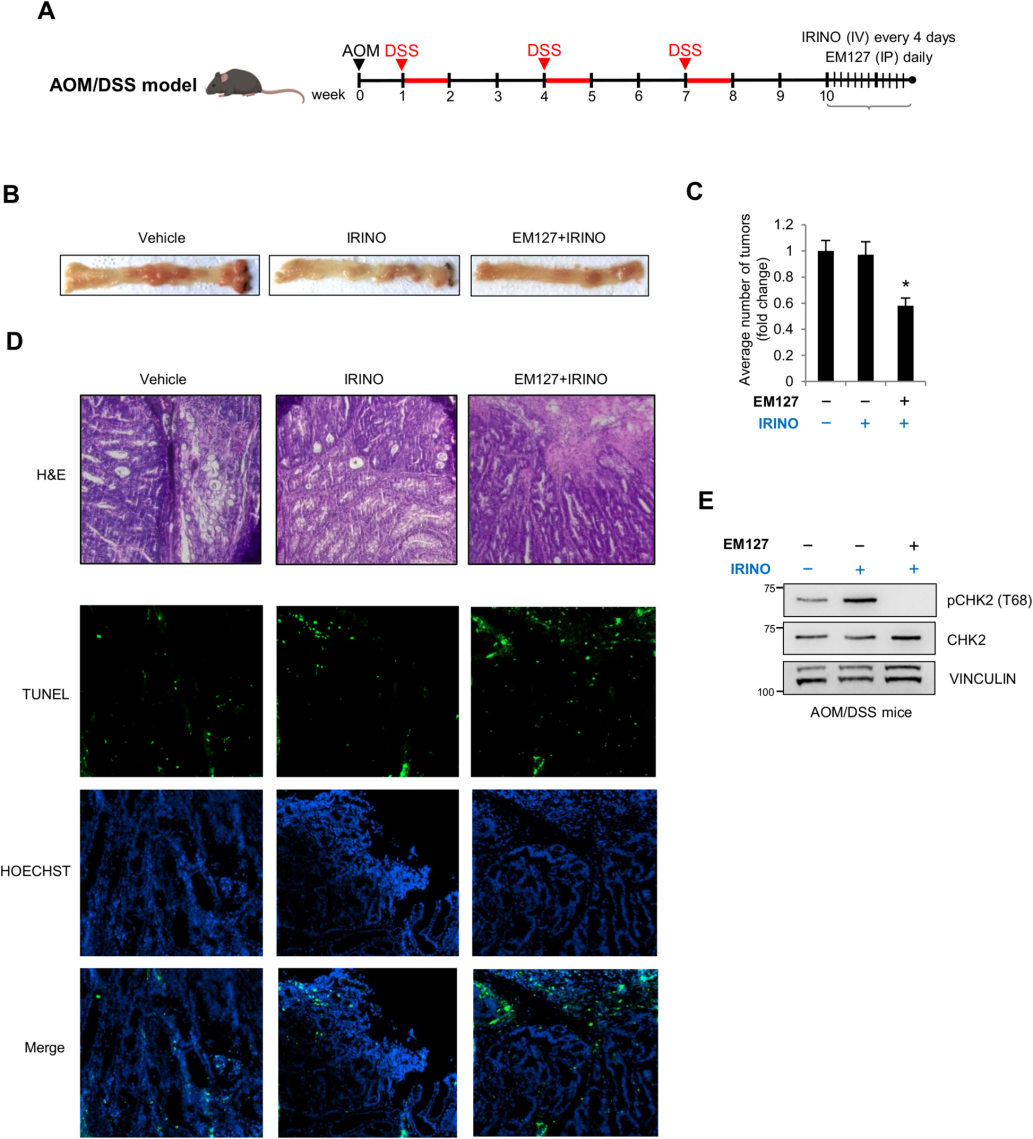
Targeting SMYD3 in an AOM/DSS murine model to circumvent chemoresistance. **A** Treatment scheme of AOM/DSS mice. **B**-**E** Examination of explanted tissues showing tumor formations (**B**), quantification of tumor number (**C**), hematoxylin and eosin and TUNEL staining with nuclei counterstained with Hoechst (blue) of colon sections (**D**) and immunoblot analysis of the phosphorylation levels of CHK2 (at T68) (**E**) from AOM/DSS mice treated or not with irinotecan (20 mg/kg) or a combination of EM127 (20 mg/kg) + irinotecan (20 mg/kg). ACTIN was used as a loading control. *p < 0.05 combined treatment vs single treatment. AOM = azoxymethan; DSS = dextran sodium sulfate; IRINO = irinotecan; IP = intraperitoneal; IV = intravenous; H&E = hematoxylin and eosin

Two weeks after the last cycle of DSS, mice were divided into three groups, which were subjected to daily intraperitoneal injections of EM127@HSA or the vehicle, combined or not with irinotecan administration by intravenous injection once every 4 days. After 12 days of treatment (Fig. 7A), the animals were sacrificed and the explanted tissues were analyzed (Fig. 7B). Interestingly, EM127 in combination with irinotecan significantly reduced the number of tumors compared with irinotecan alone (Fig. 7C). Analysis of hematoxylin and eosin-stained colon sections from mice treated with irinotecan in combination or not with EM127 revealed the presence of high-grade intramucosal carcinomas with low amount of differentiated mucus-secreting cells in vehicle and irinotecan-treated mice, whereas animals treated with both drugs displayed adenomas and moderate-grade lesions with signs of inflammation and apoptosis, which together indicate a strong regression (Fig. 7D). Moreover, evaluation of apoptotic cells by TUNEL staining revealed a significant increase in apoptosis induction in tumor sections from EM127/irinotecan-treated mice compared with animals treated with irinotecan alone or the vehicle (Fig. 7D).

In addition, since SMYD3 has been implicated in CHK2 phosphorylation (Fig. 5A, B), which is a marker of resistance and has previously been detected in resistant RC tissues (Fig. 5C), we analyzed its levels in AOM/DSS-induced mice tumor samples treated with irinotecan in combination or not with EM127. Our analysis revealed that SMYD3 inhibition with EM127 reduced phospho-CHK2 levels (Fig. 7E), confirming the involvement of SMYD3 activity in promoting chemoresistance.

Of note, EM127 improved the effect of currently used CHTs in preclinical cancer systems, enhancing tumor sensitivity to treatment. Moreover, these data validate in vivo the combined use of SMYD3is and CHTs as a promising approach to chemosensitize CRC.

## Discussion

The maintenance of genome integrity is crucial for cancer progression [46]. The goal of using exogenous agents such as CHTs is to jeopardize cancer genome stability by inducing DNA damage and therefore cell death [47]. Generally, CHTs are used in patients with different cancer stages either as neoadjuvant or as adjuvant therapy after surgery. However, a large number of patients may experience chemotherapy failure due to treatment resistance [48]. In patients with stage II or III CRC, adjuvant chemotherapy is commonly administered after surgical resection based on clinicopathological risk factors [49]. Moreover, treatment with CHTs in combination with metastasis resection is generally considered in patients with stage IV CRC or relapsed CRC and oligometastatic state of disease [49]. However, 20–30% of patients with stage II or III CRC and 60–70% of patients undergoing oligometastasis resection relapse with a tumor that is nonresponsive to further CHTs as a result of treatment resistance [50]. In patients with locally advanced rectal cancer (LARC), the standard treatment consists of neoadjuvant chemoradiotherapy plus surgery followed by optional adjuvant chemotherapy [51]. However, 30–35% of LARC patients develop distant metastasis, and compliance rates with adjuvant chemotherapy are highly inconsistent (25–75%) [51]. In patients with GC presenting with local or distant metastasis at diagnosis, chemotherapy is used as an elective treatment. However, 23 to 45% of these patients develop resistance to CHTs, which leads to poor survival [52]. In TNBC patients, chemotherapy treatment is the standard of care, but approximately 30–50% of these patients develop resistance [53].

Drug resistance is a multifactorial process involving various interrelated or independent mechanisms. Among them, cancer cells primarily counteract CHT-induced DNA damage by activating DDR signaling pathways, which orchestrate the detection and repair of DNA lesions and allow cell survival [54]. Epigenetic events are potential drivers of acquired cancer drug resistance by modulating these mechanisms. Previous reports have suggested that epigenetic adaptation to treatment contributes to the selection of cells with a resistant phenotype. Therefore, targeting epigenetic regulators represents an effective strategy to reverse drug resistance [55].

Multiple drug combinations have been developed in the last few years, which have improved response rates and overall survival, involving chemotherapy regimens in combination with novel targeted agents [4]. It is known that DDR deficiency leads to tumor sensitivity to treatment and, on the other hand, aberrant activation of DDR proteins in cancer is strongly correlated with resistance to genotoxic agents [56]. Thus, DDR inhibitors are potential candidates for cancer treatment in combination with CHTs. Davis et al. recently showed that the ATM inhibitor AZD0156 in combination with irinotecan and 5-fluorouracil leads to tumor growth inhibition in CRC preclinical models, enhancing the limited effect of AZD0156 alone [57]. Furthermore, a randomized phase 2 study in platinum-resistant high-grade serous ovarian cancer patients showed that addition of the ATR inhibitor berzosertib to gemcitabine provided benefits in terms of progression-free survival [58]. In addition, a novel class of RAD51 inhibitors has been recently described to enhance the antitumor effect of CHTs, showing strong synergy when combined with cisplatin [8].

Thus, based on the emerging role played by the methyltransferase SMYD3 in the regulation of DDR by promoting HR repair and hence cancer cell genomic stability [20], we performed an in-depth analysis of SMYD3 activity in cancer response to chemotherapy. The generation of an oxaliplatin-resistant CRC cell line and the analysis of patients’ residual gastric or rectal tumors that were resected after neoadjuvant therapy revealed a significant increase in SMYD3 protein levels in resistant samples. On the other hand, SMYD3-KO models, such as cells and xenograft mice models, revealed that SMYD3 genetic ablation reduces cancer cell resistance to CHTs, which is restored by exogenous expression of the WT protein, while the catalytically inactive form fails to do so. Moreover, we showed that SMYD3 activity is crucial for cancer cell DDR response to chemotherapy and mediates the repair of CHT-induced DSBs by methylating ATM and therefore activating the ATM-CHK2-p53 cascade. The propagation of this cascade promotes an efficient HR repair process to restore DNA and thus contributes to cancer cell chemoresistance. These data may pave the way for the design of new therapeutic strategies focused on targeting SMYD3 to sensitize cells to chemotherapy. Importantly, we found that SMYD3 pharmacological inhibition with the novel compound EM127 reverses chemoresistance, making cancer cells more sensitive to CHT exposure. Intriguingly, EM127 combined with CHTs showed a synergistic effect and induced apoptosis in 3D cellular and mice models. Thus, our findings identify EM127 as the most promising targeted covalent SMYD3i, being the first example of a second-generation potent selective inhibitor that can provide a long-lasting pharmacological action. As such, it may be considered for future therapeutic applications to treat SMYD3-positive tumors in combination with current treatments. Moreover, our data on preclinical models revealed that the combination of novel SMYD3is with chemotherapy may be an effective personalized approach for resistant cancers.

Interestingly, SMYD3 does not appear to be essential for normal development, as previously shown in SMYD3-KO mice [24, 25, 59], while it is overexpressed in a wide variety of cancers and is therefore involved in the development of these malignancies [16, 17, 60]. As such, treating SMYD3-overexpressing tumors with SMYD3is may help increase susceptibility to chemotherapy, while avoiding the side effects on normal cells that have been observed in clinical trials of other DDR inhibitors [61]. Moreover, the broad potential of SMYD3 inhibition is strengthened by our previous analysis of publicly available human cancer data from The Cancer Genome Atlas (TCGA) dataset indicating that SMYD3 mRNA levels have been found increased in around 30% of colorectal, pancreatic, and breast tumors [20].

While further research is needed to test this new therapeutic approach in clinical settings, the breadth of data obtained in cellular, 3D tumorsphere and in vivo mice models indicate that it may represent a powerful tool not only to overcome cancer resistance to existing drugs but also to reduce their dose and side effects. Moreover, it may allow the treatment of cancers not usually responding to common therapies.

## Materials and methods

### Cell lines

The HCT116, HT29, MDA-MB-231, SW480, CaCO2, HCC70, MCF7, AGS, and NCI-N87 cell lines were purchased from ATCC. The MDA-MB-231-SMYD3-KO and HCT116-SMYD3-KO cell lines were generated using the CRISPR/Cas9 technology. The U2OS cell line was kindly provided by Prof. Jeremy Stark.

The HCT116-OXA-R cell line was established from the HCT116 parental cell line by continuous exposure to oxaliplatin with stepwise increasing concentrations ranging from 1 μM to 10 μM over approximately 6 months. The CRC cell lines HCT116, HT29, SW480, and CaCO2, and the BC cell lines MDA-MB-231 and MCF7, and the U2OS DR-GFP cell line were cultured in DMEM high glucose without pyruvate (41965–039, Gibco) with 10% FBS (A5256701, Gibco) and 100 IU/ml penicillin–streptomycin (15140–122, Gibco). The BC cell line HCC70 and the GC cell lines AGS and NCI-N87 were cultured in RPMI high glucose without pyruvate (21875–034, Gibco) with 10% FBS (A5256701, Gibco) and 100 IU/ml penicillin–streptomycin (15140–122, Gibco). All cell lines were tested to be mycoplasma-free (117048; Minerva Biolabs) multiple times throughout the study. All cell cultures were maintained in a humidified incubator at 37 °C and 5% CO2.

### Clinical data

Gastric and rectal cancer tissues were obtained from 11 patients treated with neoadjuvant chemotherapy and successively subjected to surgical resection, in accordance with the ethical standards of the Institutional Committee on Human Experimentation after informed consent.

### Chemicals

All commercially available reagents were used without further purifications unless otherwise indicated. BCI-121 (SML1817), oxaliplatin (O9512), paclitaxel (T1912), 5-fluorouracil (F6627), doxorubicin (D1515), irinotecan (S-I1406), and azoxymethane (A5486) were purchased from Sigma-Aldrich Merck. KU60019 (S1570) and irinotecan HCl trihydrate used for animal studies (S2217) were purchased from SelleckChem. Dextran sodium sulfate (160110) was purchased from MP Biomedicals. EM127 was obtained through the straightforward procedure described in Parenti et al. [22]. For nanoparticle formulation, human serum albumin (HSA) (A1653) and α-tocopherol (T3251) were purchased from Sigma-Aldrich Merck. The hydrodynamic diameter and polydispersity index (PDI) of nanoparticles in aqueous solution were determined by DLS analysis at 25 °C using a NanoBrook Omni Particle Size Analyzer (Brookhaven Instruments Corporation) equipped with a 35 mW red diode laser (nominal wavelength 640 nm). Electrophoretic mobility, i.e., ζ-potential, was measured at 25 °C using the same instrument.

For each chemical, doses and treatment duration are indicated in the Figure legends.

### EM127@HSA nanoparticles: preparation

HSA was dissolved in milliQ water to achieve a final concentration of 2.5 mg/mL. The solution was vigorously stirred for 5 min and then filtered using a Chromafil® Xtra RC-45/13 0.45 μm syringe filter (SLGP033RB Sigma-Aldrich, Merck). EM127 (3.6 mg/mL) and α-tocopherol (15 mg/mL) ethanolic stock solutions were then mixed and slowly added via syringe to the HSA solution to achieve a final EM127/HSA ratio of 15% w/w and α-tocopherol/HSA ratio of 3% w/w. Sudden opalescence indicated nanoparticle formation. Then, the mixture was further vigorously stirred for 10 min at room temperature, and 200 µL of the solution were withdrawn and diluted in a cuvette with 1.6 mL of milliQ water to perform the dynamic light scattering (DLS) analysis. EM127@HSA nanoparticles showed an average hydrodynamic diameter between 80 and 100 nm, with a polydispersity index (PDI) below 0.2 and a ζ -potential value of about -25 mV. The solution was then freeze-dried, producing a white powder of EM127@HSA, which was stored at 4 °C.

### EM127@HSA nanoparticles: stability studies

In vitro stability studies were performed as follows. 0.2 mL of nanoparticle solution (1–2 mg/mL) were withdrawn and diluted with 1.6 mL of the selected stability medium at 37 °C. Changes in particle size distribution were monitored by DLS in (i) water; (ii) PBS, pH 7.4; (iii) FBS 10% in PBS, pH 7.4, v/v, for 168 h. The results in terms of particle size and PDI are illustrated in Supplementary Fig. 3A and B respectively. In water and PBS (blue and green lines, respectively), the nanoparticles showed no significant changes in hydrodynamic diameter and polydispersity values. Conversely, in FBS (red line), the nanoparticles showed a gradual and steady increase in diameter until stabilizing at around 225 nm at day 4 of observation, which can be attributed to the interaction with serum proteins; however, no visible aggregation phenomena were observed, confirming their outstanding colloidal stability over time.

### Plasmids

The FLAG-CMV2-SMYD3-WT and FLAG-CMV2-SMYD3-F183A constructs were generated as previously described [62] and kindly provided by Dr. Giuseppina Caretti. The pCBAsceI plasmid (26477) was purchased from Addgene. The pGL3 promoter vector (E1751) was purchased from Promega.

### Cell transfection and RNA interference

HCT116-SMYD3-KO cells were transiently transfected with mammalian expression plasmids using Lipofectamine 3000 (L3000015, Thermo Fisher Scientific) according to the manufacturer’s instructions. For RNA interference, HCT116 cells were transfected with 5 nM validated siRNAs directed against SMYD3 (130398, Life Technologies) by using the HiPerfect reagent (301704, QIAGEN) according to the manufacturer’s instructions. Silencer™ Select Negative Control (4390843, Life Technologies) was used as control.

### CRISPR/Cas9 system

The MDA-MB-231-SMYD3-KO cell line was obtained as previously described [20]. The TrueCut Cas9 Protein V2 and SMYD3 TrueGuide gRNA (CRISPR1032607, CRISPR 1032618, Thermo Fisher Scientific) were transfected into the HCT116 cell line using Lipofectamine CRISPRMAX Transfection Reagent (CMAX00001, Thermo Fisher Scientific) according to the manufacturer’s instructions. After 48 h, isolation of clonal populations of HCT116 cells was performed with agarose-based cloning rings (C1059, Sigma-Aldrich, Merck). Cell clones were tested for site-specific loss of function alterations by PCR, using SMYD3 gRNA sequencing FW 5'-AGCCCGTGAGACGCCCGCTGCTGG and SMYD3 gRNA sequencing RV 5'-GAAAAGTTCGCAACCGCCAA primers. Sequencing products were purified using the Dye Ex 2.0 Spin Kit (63204, QIAGEN) and sequenced on an ABI PRISM 310 Genetic Analyzer (Applied Biosystems).

### 3D models

24-well ultra-low attachment plates (3473, Corning) were used for culturing AGS, MDA-MB-231, HCT116, and HCT116-SMYD3-KO 3D tumorspheres. 3D cultures were maintained in DMEM/F12 Advanced (12634010, Gibco) supplemented with 6 mg/mL Glucose (G8769, Sigma-Aldrich, Merck), 2 mM L-Glutamine (25030081, Gibco), 10 ng/mL bFGF (F0291, Sigma-Aldrich, Merck), 20 ng/mL EGF (E9644, Sigma-Aldrich, Merck), B27 supplement (12587010, Gibco), and N-2 supplement (17502048, Gibco). An inverted phase contrast microscope was used to observe the morphology and growth of 3D tumorspheres.

### Immunoblotting

Whole-cell extracts were obtained from cells collected and homogenized in lysis buffer (50 mM Tris–HCl pH 7.4, 5 mM EDTA, 250 mM NaCl, and 1% Triton X-100) supplemented with protease and phosphatase inhibitors (78443, Thermo Fisher Scientific). Murine tissue specimens were lysed in T-PER Tissue Protein Extraction Reagent (78510, ThermoFisher Scientific) using gentleMACS M Tubes (130–093-236, Miltenyi Biotec) and homogenized on the gentleMACS Dissociator (130–093-235, Miltenyi Biotec) with the Protein_01 program, according to the manufacturer’s instructions. 20–40 µg of protein extracts from each sample were denatured in 5 × Laemmli sample buffer and subjected to 7.5% precast TGX Stain-Free polyacrylamide gel (4568024, Bio-Rad Laboratories) electrophoresis. Proteins were electrotransferred onto nitrocellulose membrane and blocked with 1 × PBS with 1% casein (1610783, Bio-Rad Laboratories) for 40 min at room temperature for immunoblot analysis. Primary antibodies used: β-ACTIN (3700, Cell Signaling Technologies), CHK2 (6334, Cell Signaling Technologies), phospho-CHK2 Thr68 (2197, Cell Signaling Technologies), FLAG M2 (F1804, Sigma-Aldrich, Merck), cleaved PARP (5625, Cell Signaling Technologies), PARP (9542, Cell Signaling Technologies), p53 (2527, Cell Signaling Technologies), phospho-p53 Ser15 (9286, Cell Signaling Technologies), SMYD3 (12859, Cell Signaling Technologies), VINCULIN (13901, Cell Signaling Technologies). Rabbit IgG HRP and mouse IgG HRP (NA934V and NA931V, respectively, GE Healthcare) were used as secondary antibodies and revealed using the ECL-plus chemiluminescence reagent (RPN2232, GE Healthcare). Densitometric evaluation was performed using ImageLab software (Bio-Rad Laboratories).

### Annexin V staining

1 × 10^6^ cells were cultured in 6-well plates for 72 h at 37 °C, 5% CO2, with complete medium. After 24 h, cells were pre-treated or not for 48 h with EM127 and then treated or not with CHTs and/or with EM127 for another 24 h. 2 × 10^4^ cells/plate were collected and resuspended in 1X PBS-1% FBS, then the Muse Annexin V and Dead Cell reagent (MCH100105, Luminex) was added to each tube. Cells were incubated at room temperature for 20 min in the dark. Flow cytometry was performed using the Guava Muse Cell Analyzer (Luminex). Cells were considered apoptotic if they were Annexin V + /PI- (early apoptotic) and Annexin V + /PI + (late apoptotic). Each analysis was performed by evaluating at least 2000 events using the assay-specific software module included in the Guava Muse Cell Analyzer instrument.

### DNA damage assay

1 × 10^6^ cells were cultured in 6-well plates for 72 h at 37 °C, 5% CO2, with complete medium. After 24 h, cells were pre-treated or not for 48 h with EM127 and then treated or not with CHTs and/or with EM127 for another 24 h. 2 × 10^4^ cells/plate were collected and resuspended in 1X assay buffer, then the Muse Multi-Color DNA Damage Kit (MCH200107, Luminex) was added to each tube. Cells were incubated with the antibody working cocktail solution at room temperature for 30 min in the dark. Flow cytometry was performed using the Guava Muse Cell Analyzer (Luminex). The kit simultaneously detects by flow analysis the phosphorylation state of two important indicators of DNA damage, ATM and histone H2A.X, in the cell population. Each analysis was performed by evaluating at least 1000 events using the assay-specific software module included in the Guava Muse Cell Analyzer instrument.

### Luciferase NHEJ repair reporter assay

The pGL3 vector was linearized using the HindIII enzyme (R3104, New England Biolabs) to induce DSBs. HCT116 cells, pre-treated with EM127 for 24 h or untreated, were transfected with 500 ng of either the linearized plasmid or the uncut pGL3 control plasmid using Lipofectamine 3000 (L3000015, Thermo Fisher Scientific), along with 10 ng of the Renilla luciferase vector as a transfection efficiency control. Luciferase activity was assessed 24 h post-transfection using the Dual Luciferase Reporter Assay Kit (E1910, Promega). Firefly luciferase activity in each sample was normalized to the Renilla luciferase signal. The percent reactivation of NHEJ was calculated by normalizing the linearized pGL3 signal to the uncut pGL3 control signal. Data are presented as relative repair efficiencies, where the proportion of reactivation from the experimental condition (EM127 treatment) is normalized to the control condition.

### DR-GFP reporter assay

U2OS DR-GFP cells were seeded in chamber slides and after 24 h they were pre-treated or not with EM127 for 2.5 h, then transfected with pCBASceI vector using Lipofectamine 3000 transfection reagent (L3000015, Thermo Fisher Scientific) according to the manufacturer’s instructions. The restriction enzyme I-SceI cuts the reporter plasmid and initiates GFP expression when the damage is repaired by HR. After 24 h, nuclei were visualized by staining with DAPI (D9542, Sigma-Aldrich, Merck); GFP-positive cells were detected and scored by fluorescence microscopy on a Zeiss Axio Observer fluorescence microscope, using a 10 × magnification objective.

### Colony formation assay

Cells were cultured in 6- or 12-well plates in the presence or absence of the indicated drugs. After treatment, media were discarded and cells were washed twice with 1X PBS. Cells were fixed with 4% paraformaldehyde for 20 min and then stained with Crystal violet solution (80299, Liofilchem). Cells were washed with water several times to remove excess of Crystal violet. Plates were dried at room temperature. Percent cell growth inhibition at each concentration was quantified by densitometric evaluation using ImageJ software.

### Cellular assays

Cell proliferation was determined using the CellTiter 96 Aqueous One Solution Cell Proliferation Assay (G3582, Promega) according to the manufacturer’s instructions. Briefly, cells were seeded into 96-well plates one day before treatment. Cells were pre-treated or not for 48 h with BCI-121 or EM127 and then treated or not with CHTs and/or with BCI-121 or EM127 for another 24 h. Then, 10 μl of the CellTiter 96 Aqueous One Solution were added to each well and incubated at 37 °C in a humidified incubator for up to 1 h. The luminescent signal was read using a SPECTROstar Omega microplate reader (BMG Labtech). The proliferation index was calculated as the ratio of the absorbance of treated cells to the absorbance of control cells**.**

Cell death was assessed by trypan blue count. Briefly, supernatants (containing dead/floating cells) were collected. Cell pellets were resuspended in 1X PBS and 10 μl were mixed with an equal volume of 0.01% trypan blue solution (T8154, Sigma-Aldrich, Merck). Viable cells (unstained, trypan blue-negative cells) and dead cells (stained, trypan blue-positive cells) were counted with a phase-contrast microscope, and the percentage of dead cells was calculated.

For live/dead assays, 3D tumorspheres were pre-treated or not for 48 h with EM127 and then treated or not with CHTs and/or with EM127 for another 24 h. After treatment, tumorspheres were stained using the LIVE/DEAD® Cell Imaging Kit (R37601, Thermo Fisher Scientific) according to the manufacturer’s instructions. ZEN microscopy software was used to quantify the average fluorescence brightness. Digital image acquisition was assessed with a on a Zeiss Axio Observer fluorescence microscope using a 5 × and 10 × magnification objectives.

### Immunohistochemistry

Tissue specimens were fixed in 4% buffered formalin and embedded in paraffin. Sequential sections (4 μm) were cut, stained with hematoxylin and eosin (H&E), and used for morphological studies and immunohistochemical analysis. Sections were dewaxed and rehydrated in dH_2_O. Endogenous peroxidase activity was blocked by incubation in 3% hydrogen peroxide for 10 min. Then, sections were mounted on Apex Bond IHC Slides (3800040, Leica Biosystems) and used for immunohistochemical analysis. Immunohistochemical staining procedures were carried out on a BOND III automated immunostainer (Leica Biosystems), from deparaffinization to counterstaining with hematoxylin, using the Bond Polymer Refine Detection Kit (DS9800, Leica Biosystems). Then, sections were incubated overnight with the primary antibody: anti-SMYD3 (ab183498, Abcam, 1:200 dilution) and anti-phospho-CHK2 (2197, Cell Signaling Technologies, 1: 100 dilution). Antigen retrieval was performed using the BOND Epitope Retrieval Solution 2, a ready-to-use EDTA-based pH 9 reagent (AR9640, Leica Biosystems). Negative controls were used in each experiment. Anti-SMYD3 and anti-phospho-CHK2 immunoreactivity was evaluated in a blinded manner by two independent pathologists, who used a semiquantitative approach to score the percentage of positive stained cells and the intensity of the staining (0: absent, 1: mild and focal, 2: moderate, 3: intense and diffuse). Images were acquired using a Zeiss Axio Observer Z1 optical microscope (Carl Zeiss).

### Methylation luminescent assay

Analysis of SMYD3 methylation activity was performed using a luminometric methylation assay, MTase-Glo™ Methyltransferase Assay (V7601, Promega). SMYD3 active protein was expressed and purified as previously described [63]. Briefly, SMYD3 active protein (500 ng) was assayed in a methylation reaction buffer containing 50 mM Tris (pH 8), 4 mM MgCl_2_, 0.2% Tween-20, 2 mM DTT, 200 mM SAM, and 500 ng of ATM protein (14–933, Sigma-Aldrich, Merck) in a final volume of 20 μL. Histone H4 Peptide (1–21) (650 ng, H13-58 SignalChem) was used as a positive control. The reaction was incubated overnight at 30 °C. Then, 5 μL of 5 × MTase-Glo reagent was added to convert SAH to ADP. Next, MTase-Glo™ Detection Solution was added to convert ADP to ATP, which was determined by a luciferase/luciferin reaction. The generated luminescence was measured using a SPECTROstar Omega microplate reader (BMG Labtech). Each data point was collected in triplicate. For enzymatic inhibition, SMYD3 was pre-incubated in the presence of BCI-121 (100 μM) or EM127 (5 μM) in the assay buffer for 8 h. Then, methylation assay was performed as reported above.

### Immunofluorescence and foci counting

Cells were seeded on glass coverslips, treated as indicated for each experiment, and then fixed with 4% paraformaldehyde, and permeabilized using 0.1% Triton X-100. Coverslips were incubated with the indicated primary antibodies and then with Alexa Fluor 488 (A-11094, Thermo Fisher Scientific) and 647 (A-32728, Thermo Fisher Scientific) secondary antibodies; nuclei were counterstained using DAPI (D9542, Sigma-Aldrich, Merck). Slides were sealed using ProLong Diamond Antifade Mountant (P36961, Thermo Fisher Scientific). Images were acquired using a Zeiss fluorescence microscope. Primary antibodies: SMYD3 (ab183498, Abcam), 53BP1 (NB100-304, Novus Biologicals), MRE11 (4847, Cell Signaling Technologies), RAD51 (ab133534, Abcam), and RPA32/RPA2 (35869, Cell Signaling Technologies). Densitometric evaluation was performed using ZEN microscopy software. Foci were scored by fluorescence microscopy using a 63 × magnification objective and digital image acquisition on a Zeiss Axio Observer fluorescence microscope.

### In vivo studies

The HCT116-xenografted mice model was established at the Biogem Animal House in Ariano Irpino (Avellino, Italy) under the National Academy of Sciences guidelines. 10 × 10^6^ HCT116-SMYD3-KO cells or HCT116 parental cells were injected subcutaneously into the flanks (0.2 mL per flank, serum-free DMEM culture medium) of 5 to 6-week-old CD1 immunodeficient female nude mice (Charles River Laboratories International, Inc.). During the study, each mouse was given drinking water ad libitum and a complete pellet diet. Mice were monitored daily for clinical signs and mortality, and their body weight was assessed every 2–3 days. The tumor volume was measured every 2–3 days using the following formula: volume (mm^3^) = (width)^2^ × length × 0.5 with Mitutoyo forceps. When the tumor volume reached 100 mm^3^, mice were randomized into two treatment groups. Mice were treated every 4 days for 12 days with 20 mg/kg of irinotecan by intravenous injection (*n* = 20) or the vehicle (DMSO) alone (*n* = 20). At the end of the study, mice were sacrificed by cervical dislocation, and the tumor masses were photographed and collected.

The chemical-induced colitis-associated carcinogenesis mice model was established at the IRCCS “S. De Bellis” Animal House in Castellana Grotte (Bari, Italy). C57BL/6 mice (*n* = 36) were injected intraperitoneally with 12 mg/kg of AOM. Then, 2% DSS was given in their drinking water over a week, followed by two weeks of regular water. This cycle was repeated three times. Two weeks after the last round of DSS, mice were treated for 12 days with a combination of 20 mg/kg of EM127@HSA dissolved in physiologic water and 20 mg/kg of irinotecan (*n* = 12) or with irinotecan alone (*n* = 12) or with the vehicle (DMSO) alone (*n* = 12). For these treatments, EM127@HSA was administered daily by intraperitoneal injection, irinotecan was administered by intravenous injection every 4 days, and DMSO was administered by intravenous injection every 4 days.

All tissues were fixed overnight in 10% formalin and embedded in paraffin. The procedures involving animals were conducted in conformity with the institutional guidelines that comply with national and international laws and policies. For AOM/DSS–treated animals, welfare was recorded every 2–3 days during the DSS/control treatment regimen by use of a customized score sheet for bodyweight, appearance and behavior, and rectal prolapse (Supplementary Table 1). Assessment of appearance and behavior included evaluation of coat, posture and the stool consistency. Animals exhibiting symptoms were kept under close observation.

### Methylene blue staining

Methylene blue was used to stain and score the number of tumors. The colon was excised from the anus to the cecum, rinsed, and flushed with ice-cold PBS to remove any intestinal contents and then slit open longitudinally. Next, the colon was fixed flat between two PBS-soaked filter papers held together with staples. The flat-fixed colon was then stored in 10% neutral buffered formalin for at least 24 h before staining and removed from the filter paper. Any remaining fat on the muscularis side of the colon was carefully removed with forceps, and then the colon was transferred to a new beaker filled with distilled water. After 1 min, the colon was transferred to another beaker containing a 70% ethanol solution for 45 min. Next, the colon was moved to a beaker containing 0.2% methylene blue (M9140, Sigma-Aldrich, Merck), stained for 5–10 s, and transferred to a new beaker filled with distilled water to wash off excess methylene blue.

Colon specimens were mounted on a microscope slide and observed under a Carl Zeiss inverted microscope equipped with a digital color camera. Once scored, specimens were paraffin-embedded.

### TUNEL assay

The TUNEL assay was performed using the Click-iT Plus TUNEL Assay kit (C10618, Thermo Fisher Scientific) according to the manufacturer’s instructions. Briefly, tissue sections were deparaffinized, fixed in 4% paraformaldehyde, and permeabilized with Proteinase K. Sections were then soaked in TdT reaction buffer at 37 °C for 10 min. Next, the TdT reaction buffer was discarded, and sections were incubated at 37 °C for 60 min with the TdT reaction mixture. Subsequently, sections were incubated at 37 °C for 30 min in the dark with the Click-iT Plus TUNEL reaction cocktail. Sections were then stained with Hoechst 33342 (62249, Thermo Fisher Scientific) and examined by fluorescence microscopy.

### Quantification and statistical analysis

Data were analyzed and plotted using Microsoft Excel, GraphPad Prism, Combenefit, ImageJ, or ZEN microscopy software. Statistical analysis was performed using Student’s t-test. Differences were considered significant when the *p*-value was < 0.05. At least three independent experiments were performed for each assay.

### Supplementary Information

Supplementary Material 1: Supplementary Figure 1. (A) Immunoblot analysis of PARP and cleaved PARP by using two different antibodies in HCT116 cells treated with BCI-121 (100 μM) or EM127 (5 μM) and/or doxorubicin (1 μM) as described in Figure 1A. ACTIN was used as a loading control (left panel). Ratio of cleaved PARP to total PARP determined by measuring the optical density of the immunoblot bands (right panel). (B) Flow cytometry analysis of Annexin V staining in HCT116 cells treated with different doses (10, 30 μM) of irinotecan for different times (24, 48, 72 h) compared with cells pre-treated with EM127 (5 μM) for 48 h and then treated with irinotecan (10 μM) for 24 h in the presence of EM127. (C) Bliss synergy surface analysis obtained with Combenefit software of HCT116 cells treated with different concentrations of EM127 (0, 1, 2.5, 5 μM) and doxorubicin (0, 5, 10, 20 μM). (D) Quantification of apoptotic cell death by Annexin V staining in HT29, SW480, and CaCO2 cells treated as described in Figure 4A. (E, F) Flow cytometry analysis of Annexin V staining in MDA-MB-231, HCC70, and MCF7 BC cell lines (E) and AGS and NCI-N87 GC cell lines (F) pre-treated or not with EM127 (5 μM) for 48 h and then treated or not with 5-fluorouracil (10 μM) or paclitaxel (100 nM) (BC cell lines) or with paclitaxel (10 nM) or oxaliplatin (10 μM) (GC cell lines) for another 24 h in the presence of EM127. *p<0.05 treated vs. untreated. #*p*<0.05 combined treatment vs. single treatments. cl.PARP = cleaved PARP; DMSO = dimethyl sulfoxide; DOXO = doxorubicin; 5-FU = 5-fluorouracil; IRINO = irinotecan; OXA = oxaliplatin; PTX = paclitaxel. Supplementary Figure 2. (A) Immunoblot analysis of the phosphorylation levels of CHK2 (at T68) and p53 (at S15) in CaCO2 and HT29 cells pre-treated or not with EM127 (5 μM) for 24 h and then exposed or not to irinotecan (10 μM) for 6 h in the presence of EM127. VINCULIN was used as a loading control. (B) Densitometric analysis of the phosphorylation levels of CHK2 (at T68) and p53 (at S15) measured by immunoblot in HCT116 cells pre-treated or not with EM127 (5, 2.5 μM) and/or KU60019 (5, 2.5 μM) for 24 h and then exposed or not to irinotecan (10 μM) for 6 h in the presence of the inhibitors. (C) Quantification of cell proliferation by CellTiter 96 Aqueous Assay in HCT116 cells pre-treated or not with EM127 (5 μM) and/or KU60019 (5 μM) for 48 h and then treated or not with irinotecan for 24 h in the presence of the inhibitors. (D) Immunostaining for 53BP1 (red) in HCT116 cells pre-treated or not with EM127 (5 μM) for 2.5 h and then exposed or not to irinotecan (10 μM) and fixed at the indicated time points. Nuclei were counterstained with DAPI (blue). (E) Flow cytometry analysis of total DNA damage in HCT116 cells pre-treated or not with EM127 (5 μM) for 12 h and then exposed or not to irinotecan (10 μM) for 6 h. Cells were stained for pATM (S1981) and γH2AX (S139) to calculate the percentage of cells with DNA damage. (F) Immunostaining for MRE11, RPA32 and RAD51 (green) in HCT116 cells pre-treated or not with EM127 (5 μM) for 2.5 h and then exposed or not to irinotecan (10 μM) for 6 h. Nuclei were counterstained with DAPI (blue).*p<0.05 treated vs. untreated. #*p*<0.05 EM127 and KU60019 combined treatment vs. single treatments. DMSO = dimethyl sulfoxide; IRINO = irinotecan. Supplementary Figure 3. Stability studies determined by DLS analysis. (A) Particle size changes of EM127@HSA nanoparticles in H_2_O pH 7.4 (blue line), in PBS pH 7.4 (green line), FBS 10% in PBS pH 7.4, v/v (red line); (B) Polydispersity index changes of EM127@HSA nanoparticles in H_2_O pH 7.4 (blue dotted line), in PBS pH 7.4 (green dotted line), FBS 10% in PBS pH 7.4, v/v (red dotted line).

Supplementary Material 2.

## Data Availability

Source data are provided with this paper. All other data supporting the findings of this study are available from the corresponding author on reasonable request.
