# Supplementary figures and images for: The novel SMYD3 inhibitor EM127 impairs DNA repair response to chemotherapy-induced DNA damage and reverses cancer chemoresistance
Source: J Exp Clin Cancer Res. 2024 May 30;43:151. doi: 10.1186/s13046-024-03078-9 (PMC11137994; doi:10.1186/s13046-024-03078-9)

A

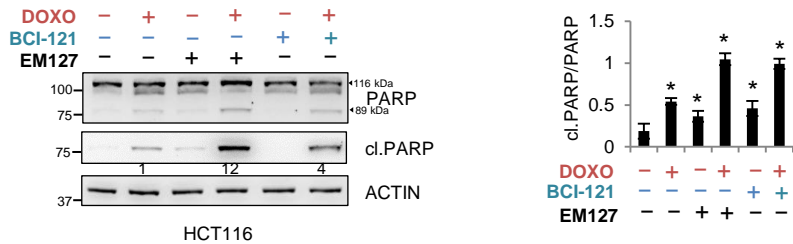

B

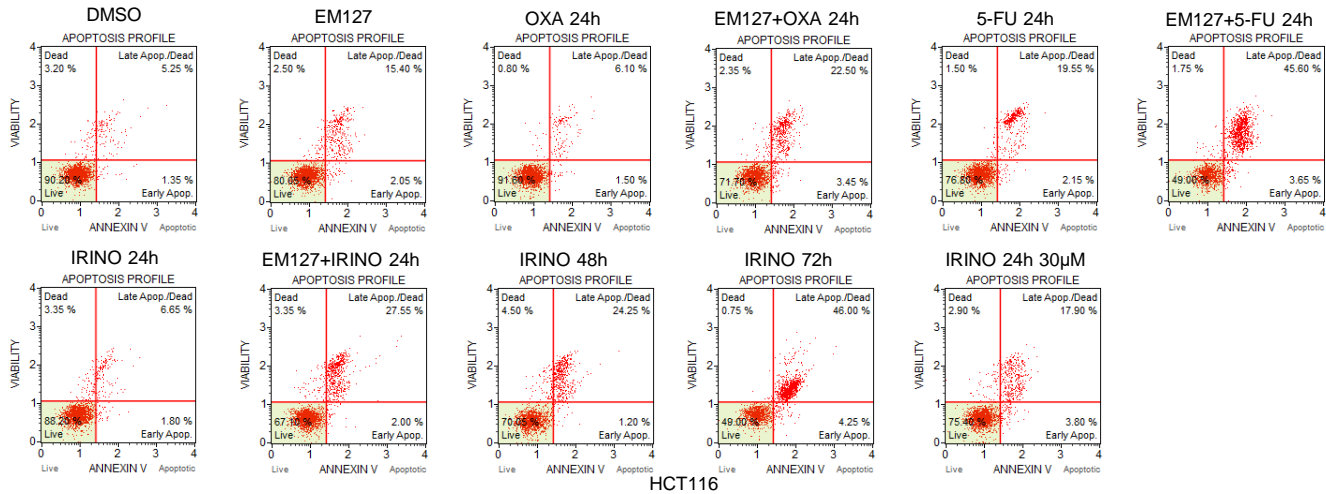

C

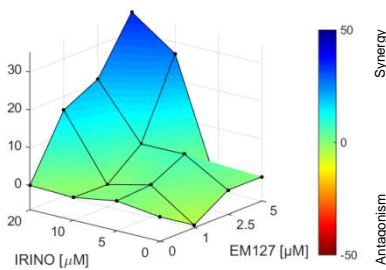

D

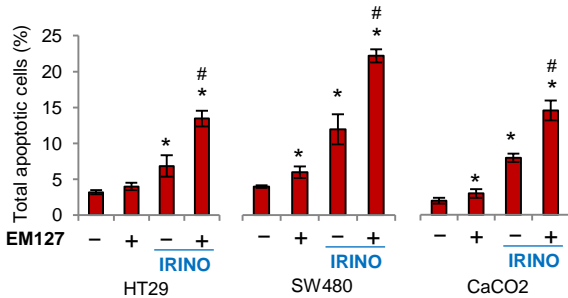

E

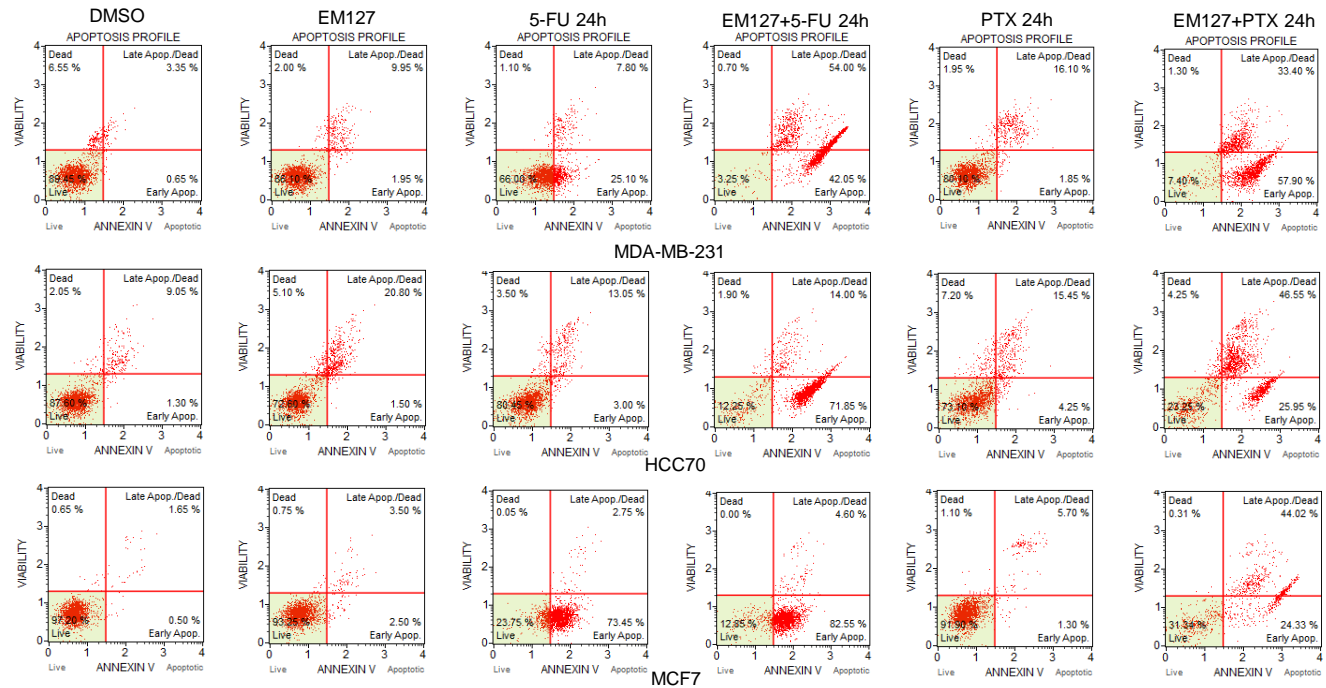

F

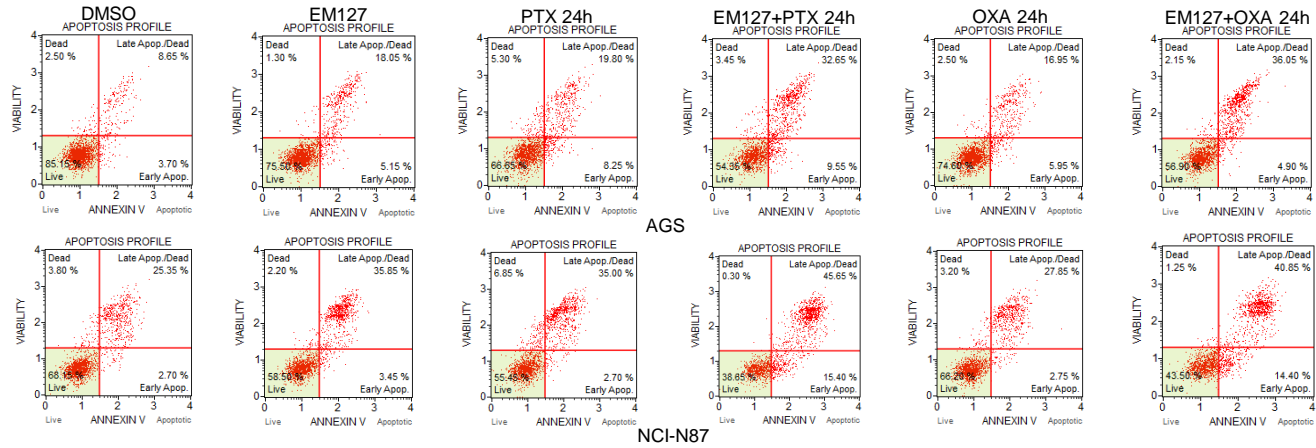

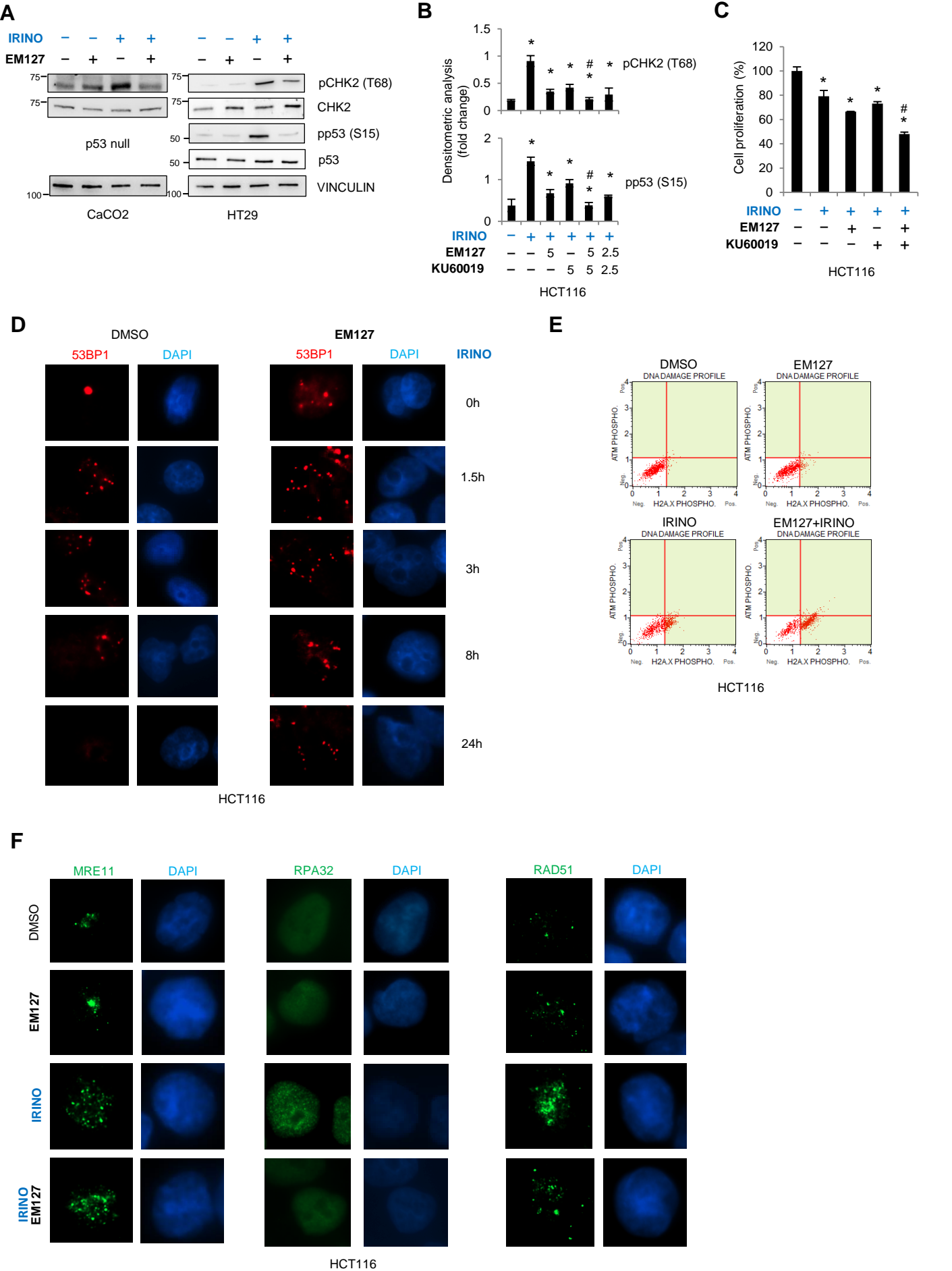

A

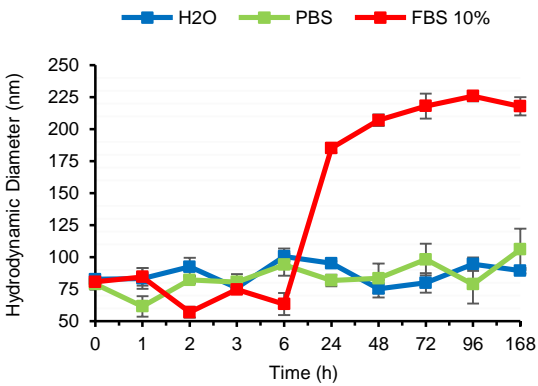

B

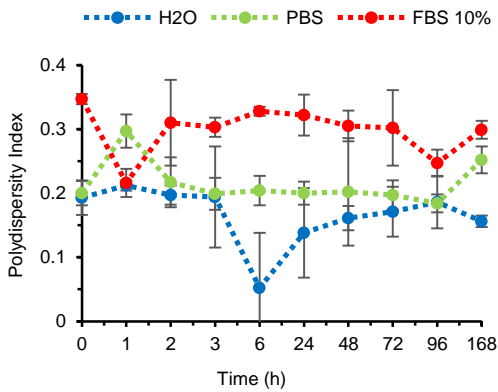

Supplement: Supplementary file 1 — Supplementary Material 1: Supplementary Figure 1. (A) Immunoblot analysis of PARP and cleaved PARP by using two different antibodies in HCT116 cells treated with BCI-121 (100 μM) or EM127 (5 μM) and/or doxorubicin (1 μM) as described in Figure 1A. ACTIN was used as a loading control (left panel). Ratio of cleaved PARP to total PARP determined by measuring the optical density of the immunoblot bands (right panel). (B) Flow cytometry analysis of Annexin V staining in HCT116 cells treated with different doses (10, 30 μM) of irinotecan for different times (24, 48, 72 h) compared with cells pre-treated with EM127 (5 μM) for 48 h and then treated with irinotecan (10 μM) for 24 h in the presence of EM127. (C) Bliss synergy surface analysis obtained with Combenefit software of HCT116 cells treated with different concentrations of EM127 (0, 1, 2.5, 5 μM) and doxorubicin (0, 5, 10, 20 μM). (D) Quantification of apoptotic cell death by Annexin V staining in HT29, SW480, and CaCO2 cells treated as described in Figure 4A. (E, F) Flow cytometry analysis of Annexin V staining in MDA-MB-231, HCC70, and MCF7 BC cell lines (E) and AGS and NCI-N87 GC cell lines (F) pre-treated or not with EM127 (5 μM) for 48 h and then treated or not with 5-fluorouracil (10 μM) or paclitaxel (100 nM) (BC cell lines) or with paclitaxel (10 nM) or oxaliplatin (10 μM) (GC cell lines) for another 24 h in the presence of EM127. *p<0.05 treated vs. untreated. #p<0.05 combined treatment vs. single treatments. cl.PARP = cleaved PARP; DMSO = dimethyl sulfoxide; DOXO = doxorubicin; 5-FU = 5-fluorouracil; IRINO = irinotecan; OXA = oxaliplatin; PTX = paclitaxel. Supplementary Figure 2. (A) Immunoblot analysis of the phosphorylation levels of CHK2 (at T68) and p53 (at S15) in CaCO2 and HT29 cells pre-treated or not with EM127 (5 μM) for 24 h and then exposed or not to irinotecan (10 μM) for 6 h in the presence of EM127. VINCULIN was used as a loading control. (B) Densitometric analysis of the phosphorylation level [file 13046_2024_3078_MOESM1_ESM.pdf]
